# BAP31 depletion inhibited adipogenesis, repressed lipolysis and promoted lipid droplets abnormal growth via attenuating Perilipin1 proteasomal degradation

**DOI:** 10.7150/ijbs.82178

**Published:** 2023-03-13

**Authors:** Xueying Wei, Liya Li, Jie Zhao, Yan Huo, Xiaodi Hu, Jingyi Lu, Jingbo Pi, Wei Zhang, Lisheng Xu, Yudong Yao, Jialin Xu

**Affiliations:** 1 Institute of Biochemistry and Molecular Biology, College of Life and Health Sciences, Northeastern University, Shenyang, 110819, Liaoning, China; 2 Institute of Microbial Pharmaceuticals, College of Life and Health Sciences, Northeastern University, Shenyang, 110819, Liaoning, China; 3 School of Public Health, China Medical University, Shenyang, 110122, Liaoning, China; 4 Department of Hepatobiliary Surgery, General Hospital of Northern Theater Command of the Chinese People's Liberation Army, Shenyang, 110016, Liaoning, China; 5 College of Medicine and Biological Information Engineering, Northeastern University, Shenyang, 110819, Liaoning, China; 6 Department of Electrical and Computer Engineering, Stevens Institute of Technology, Hoboken, NJ, 07030, USA

**Keywords:** BAP31, Perilipin1, Lipid droplet, Lipolysis, Proteasomal degradation

## Abstract

BAP31 expression was robustly decreased in obese white adipose tissue (WAT). To investigate the roles of BAP31 in lipid metabolism, adipocyte-specific conditional knockout mice (BAP31-ASKO) were generated. BAP31-ASKO mice grow normally as controls, but exhibited reduced lipid accumulation in WAT. Histomorphometric analysis reported increased adipocyte size in BAP31-ASKO mice. Mouse embryonic fibroblasts (MEFs) were induced to differentiation to adipocytes, showed reduced induction of adipogenic markers and attenuated adipogenesis in BAP31-deficient MEFs. BAP31-deficiency inhibited fasting-induced PKA signaling activation and the fasting response. β3-adrenergic receptor agonist-induced lipolysis also was reduced, accompanied by reduced free-fatty acids and glycerol release, and impaired agonist-induced lipolysis from primary adipocytes and adipose explants. BAP31 interacts with Perilipin1 via C-terminal cytoplasmic portion on lipid droplets (LDs) surface. Depletion of BAP31 repressed Perilipin1 proteasomal degradation, enhanced Perilipin1 expression and blocked LDs degradation, which promoted LDs abnormal growth and supersized LDs formation, resulted in adipocyte expansion, thus impaired insulin signaling and aggravated pro-inflammation in WAT. BAP31-deficiency increased phosphatidylcholine/phosphatidylethanolamine ratio, long chain triglycerides and most phospholipids contents. Overall, BAP31-deficiency inhibited adipogenesis and lipid accumulation in WAT, decreased LDs degradation and promoted LDs abnormal growth, pointing the critical roles in modulating LDs dynamics and homeostasis via proteasomal degradation system in adipocytes.

## Introduction

Obesity is one of the most widespread problems facing our society's health and is highly associated with type 2 diabetes, cardiovascular disease, fatty liver disease, and cancer 1. Aberrant expansion of adipose tissue occurs through an increase in adipocyte numbers (hyperplasia) or an enlargement in adipocyte size (hypertrophy) 2. Adipogenesis is the process which stores excessive energy in the form of lipid droplets (LDs). LDs are dynamic cellular organelles that store free-fatty acids (FFA) into triglycerides (TAG), and suppress lipotoxicity by preventing cell death, endoplasmic reticulum (ER) stress, and mitochondrial dysfunction 3. LDs provide a natural source of stored lipids that can be mobilized, which release FAs that can be broken down by β-oxidation for energy use. However, enlarged LDs due to lipid overloading increased adipocyte size and promoted unilocular LDs formation, and leaded to the development of obesity and insulin resistance 4. Controlling LDs abnormal growth is promised to be one of the options for preventing the development of obesity. Protein factors on LDs surface is vital for controlling LD biology and is most apparent during adipocyte differentiation.

Perilipin1 (Plin1) is one of the most important LD-associated surface proteins, involved in LDs stabilization and lipolysis by lipase and the cofactors 5. Plin1 has been reported playing a dual role in inhibiting basal or facilitating stimulated lipolysis. Signaling or agonists leads to PKA signaling activation, and induces the phosphorylation of Plin1, which promotes the translocation of Hormone-sensitive lipase (Hsl) to LDs surface and enhance TAG hydrolysis 5. Plin1-null mice exhibited enhanced basal lipolysis rate, due to the loss of the protective roles of Plin1 and the barrier for LDs. Loss-of-perilipin function resulted in lean and healthy mice, which are resistant to diet-induced obesity and insulin resistance 6. Plin1 promoted unilocular LDs formation and increased LD size through the activation of Fat-specific protein 27 (Fsp27), illustrating the functional cooperation between Plin1 and Fsp27 is required for LDs growth 7. Plin1 protein levels on LDs surface were dynamically regulated. Lipid overloading promoted LDs growth and increased perilipin protein levels via post-translationally stabilization of newly synthesized perilipins 8. Plin1 interacted with ubiquitin, and the ubiquitin-proteasome system was confirmed involved in Plin1 protein degradation 9, 10.

B-cell receptor-associated protein 31 (BAP31) is a multi-pass transmembrane protein of the ER 11, expresses ubiquitously and has been implicated in apoptosis 12, cancer development 13, ER exporting and retention 14, immune system regulation 15, protein degradation and quality control 16. Patients with BAP31 mutations suffered from motor and intellectual disabilities, dystonia, and cholestatic liver disease 17. The endothelial depletion of BAP31 attenuated LPS-induced acute lung injury via attenuating neutrophils-ECs adhesion, suggesting the important roles of BAP31 in regulating inflammatory response 18. Even ER plays an important role in lipid metabolism and BAP31 services as an evolutionarily conserved protein of the ER, relatively few research focused on lipid metabolism is available. BAP31 and ABCD1 mutation resulted in hepatomegaly and visible vacuoles in hepatocytes 19. BAP31 coupled with VAPB, VCP, FAF1, and Derlin-1, regulated the degradation of ΔF508-CFTR and lipid homeostasis, leading to the observed phenotypes of lipid abnormalities in protein folding diseases 20. Our previous publications reported that BAP31-deficiency in hepatocytes promoted SREBP1C activity and increased hepatic lipid accumulation 21, and enhanced ER-stress induced liver steatosis in mice 22. Whether BAP31 affects ER-derived LDs biology and modulates lipid accumulation in white adipose tissue (WAT) is still uncertain. Herein, the adipocyte-specific conditional knockout mice were generated. The effects on lipid accumulation and the underlying mechanisms regarding LDs growth and lipolysis were determined.

## Materials and Methods

### Mice breeding

BAP31 is an X-linked gene. BAP31^flox^ allele mice (BAP31^flox/-^) on C57BL/6 background 21 were mated with the transgenic mice expressing Cre recombinase under adiponectin promoter (adipo-Cre) control, to obtain adipo-Cre;BAP31^flox/-^ and adipo-Cre;BAP31^flox/flox^ offspring, and then were crossed with BAP31^flox/flox^ or BAP31^flox/-^allele mice, to generate adipo-Cre;BAP31^flox/-^ (specific deletion of BAP31 in adipocyte, BAP31-ASKO) and BAP31^flox/-^ (WT) mice. Male mice were used in the current study. All procedures were approved (NEU-EC-2021A036S) by the institutional review board of Northeastern University in accordance with the Guide for the Care and Use of Laboratory Animals.

### HFD feeding

WT and BAP31-ASKO mice (6-week-old) were fed with a high-fat diet (HFD. H10060, Beijing HFK Bioscience Co. Ltd.) for 14 weeks. Body weight (BW) was measured every 4 days. After exposure for 13 weeks, mice were food deprived for 16 or 5 hours, and then injected with glucose (2 mg/kg) or insulin (0.75 U/kg) solution intraperitoneally. The blood glucose at 0, 15, 30, 60, and 120 minutes were determined using a one-touch glucometer (Bioland technology, Shenzhen, China).

### Fasting treatment

WT and BAP31-ASKO mice were food deprived with free access to water for 24 hours, and then were decapitated under deep anesthesia with isoflurane. Liver and WAT were dissected. The blood samples were withdrawn. Sera were purified and stored in -80°C for future experiments.

### Hematoxylin and eosin (H/E) staining

Sections (4 μm) of paraffin-embedded WAT, BAT and liver were cut and stained with hematoxylin and eosin before histopathologic analysis. For adipocyte diameter analysis, five fields from each section were taken and totally more than 400 adipocytes were analyzed for each group.

### Serum and lipid extracts measurement

Serum glucose, TAG, FFA, cholesterol, and glycerol were measured using the kits from Nanjing Jiancheng Biomedical Company (Nanjing, China) and Solarbio Life Sciences (Beijing, China). Differentiated adipocytes or WAT (~50 mg) were lysed with PBS. Lipids were extracted with chloroform-methanol (2:1; v/v), and then were evaporated to dryness in a vacuum dryer set at 45°C for 2 hours. The lipid residue was dissolved in 100% ethanol containing 1% Triton X-100. TAG and FFA content were determined. The results were normalized with used tissue weight or cellular protein amount 23.

### Induction of adipocyte differentiation

Mouse embryonic fibroblasts (MEFs) were isolated from 13.5- to 15.5-dpc mouse embryos. MEFs and 3T3-L1 preadipocytes were cultured in DMEM containing 10% FBS. Two days post 90% confluence, MEFs were induced to differentiation to adipocytes by switching to differentiated media (10 μg/mL insulin, 1 μM dexamethasone, and 0.5 mM isobutylmethylxanthine) for the first 3 days, and then incubated with the maintaining media (10 μg/mL insulin) for the remaining days 23.

### DNA transfection and gene silencing

Full-length cDNAs encoding various mouse proteins were amplified by PCR from the cDNA of 3T3-L1 preadipocytes. cDNA encoding mouse BAP31 and Plin1 were cloned into pcDNA3.1(-) (Thermo Fisher Scientific). Plasmid DNA and siRNA were introduced into 3T3-L1 preadipocytes by using lipo8000^TM^ (Beyotime Biotechnology, Shanghai, China). For the stable cell line, 3T3-L1 preadipocytes were infected with lentivirus targeted with BAP31 for 24 hours, and then treated with 2 μg/mL of puromycin for 4-5 days to select the positive clones. The resistant cells were diluted and seeded on a 96-well plate to form a single colony. The knockdown efficiency was evaluated by immunoblotting analysis.

### Immunostaining

3T3-L1 preadipocytes cultured on coverslips were fixed and permeabilized, blocked with 10% goat serum for 1 hour and incubated with anti-BAP31 (1:200) and anti-Plin1 (1:200) antibodies at 4°C overnight, followed by incubation with the secondary antibodies (1:500) for 1 hour at room temperature. LDs were stained with Nile red (1:1000. Sigma-Aldrich. 72485) in PBS for another 10 minutes. Nucleus were stained with DAPI for 5 minutes. The fluorescence was visualized using a confocal laser scanning microscope (Leica Biosystems, Wetzlar, Germany).

### Quantitative Real-time PCR

RNA was extracted with Trizol reagent and 2 μg of total RNA was converted to cDNA. The relative mRNA levels were quantified by quantitative real-time PCR using a CFX96 Touch™ real-time PCR detection system (Bio-Rad Laboratories, CA, USA). SYBR chemistry was used and the primer sequences are listed in table S1. 18S rRNA expression was used as the loading control. Comparative cycle threshold method (ΔΔCt) was used for gene expression analysis.

### Immunoblotting analysis

WAT and adipocytes homogenates were prepared with RIPA buffer (150 mm NaCl, 50 mm Tris-HCl, 0.5% sodium deoxycholate, 1% Triton X-100, 0.1% SDS) containing freshly added protease and phosphatase inhibitors. The homogenates were resolved by SDS-PAGE, and then transferred to PVDF membrane. The membrane was blocked with 5% nonfat dry milk or 2% BSA in TBST, and then immunoblotted with the primary antibodies at 4°C overnight, followed by incubation with the secondary antibodies for 1 hour at room temperature. The sources and dilutions of antibodies are listed in table S2. GAPDH and β-Actin were used as the loading control in the individual experiment.

### Co-immunoprecipitation (Co-IP)

Cells transfected with BAP31-Flag and/or Pin1-HA constructs were lysed with IP lysis buffer (20 mM Tris (pH 7.5), 150 mM NaCl, 1% Triton X-100) with protease inhibitor cocktail (Beyotime Biotechnology). After being centrifuged at 12,000 g, the supernatants were incubated with anti-Flag and/or anti-HA antibodies overnight at 4°C with slow shaking. The protein A/G beads were added and incubated at 4°C for 3-4 hours. The beads were washed with IP buffer three times before adding the laemmli loading buffer. Proteins Co-IP were analyzed by immunoblotting analysis.

### Lipolysis rate measurement

WT and BAP31-ASKO mice (12-week-old) were injected with CL316,243 (0.1 mg/kg) intraperitoneally. One hour later, mice were sacrificed under anesthesia and sera were extracted. Serum FFA and glycerol were determined to illustrate the lipolysis rate 24. Or the epididymal and subcutaneous fat pads were dissected and minced into small pieces of about 3 mm, and then were placed in serum-free culture medium (phenol red-free DMEM containing 1 μM of CL316,243 with 1% FA-free BSA). Two hundred microliters medium was collected at 0, 1, 2, and 3 hours after CL316,243 treatment. FFA and glycerol released in the medium were measured then.

The primary mature adipocytes were isolated from epididymal and subcutaneous fat pads of WT and BAP31-ASKO (12-week-old) mice as previously described 25; and then were incubated with phenol red-free and serum-free DMEM containing 1% FA-free BSA, in the presence or absence of 10 nM of isoproterenol (ISO). FFA and glycerol released into the medium were measured at 0, 1, 2, 3, and 5 hours after ISO administration.

### Lipidomics analysis

Lipids in epididymal WAT were extracted following a modified Bligh and Dyer's method described as before 26. Lipid profiles were measured using a high-coverage targeted lipidomic approach constructed principally on HPLCMRM, with the modification of the selection of internal standards used for quantification. The lipidomic analyses were performed using an Exion LC-system coupled with a QTRAP 6500 PLUS system (Sciex). The content of the individual lipids from various classes were quantitated relative to the respective internal standard.

### Statistical analysis

Data were presented as mean ± SE. The statistical analysis was plotted using Graphpad Prism 5.0. One-way ANOVA followed by Tukey post hoc test was used to determine the significance of the individual differences. All statistical tests with *p* < 0.05 were considered as significant.

## Results

### BAP31-deficiency reduced lipid accumulation in white adipose tissue, but induced adipocyte expansion in mice

**Figure 1:**
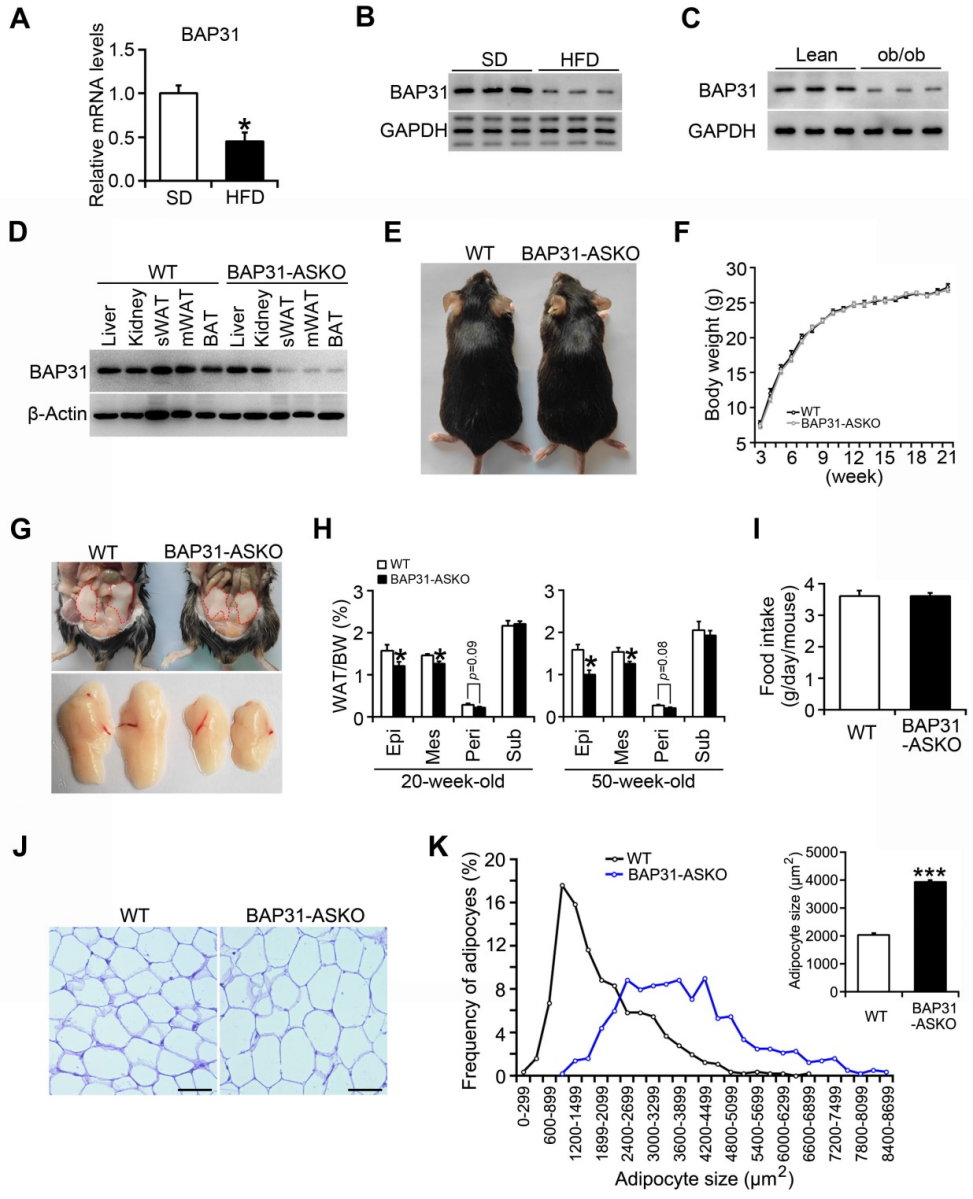
** BAP31-deficiency reduced lipid accumulation in white adipose tissue, but induced adipocyte expansion in mice. (A and B)** BAP31 mRNA and protein levels were decreased in WAT of HFD-induced obese mice. *p<0.05, compared to SD mice. **(C)** BAP31 protein levels were decreased in WAT of ob/ob mice. **(D)** BAP31 expression was depleted in adipocyte-specific BAP31 conditional knockout mice (BAP31-ASKO). sWAT: subcutaneous WAT. mWAT: mesenteric WAT. **(E)** The photos of WT and BAP31-ASKO mice. **(F)** The body weight was comparable between WT and BAP31-ASKO mice. n=9. **(G)** The representative pictures of epididymal WAT. **(H)** The organ indexes of epididymal WAT (Epi), mesenteric WAT (Mes), perirenal WAT (Peri), and subcutaneous WAT (Sub) from 20-week-old (n=8) and 50-week-old mice (n=8-10). **(I)** Food intake is comparable between WT and BAP31-ASKO mice. n=8. **(J)** The representative images of H/E staining of epididymal WAT. Scale bar=50 μm. n=4. **(K)** The frequency of adipocytes and the mean adipocyte size in epididymal WAT. *p<0.05, ***p<0.001, compared to WT mice.

BAP31 mRNA and protein levels were reduced in WAT of diet-induced obese mice (Figure 1A and 1B), and also decreased in leptin deficiency-induced obese mice (Figure 1C), pointing the reasonable roles involved in obesity. Therefore, mice with targeted deficiency in adipocytes were generated. BAP31 expression was depleted in white and brown adipose tissues (Figure 1D). The conditional knockout mice grow normally as controls. BW curve analysis also shows no significant difference between these two genotypes of mice (Figure 1E and 1F). BAP31-ASKO mice exhibited reduced epididymal, mesenteric, and perirenal WAT mass than WT controls, with no difference in subcutaneous WAT mass at 20- and 50-week-old age, suggesting that BAP31-deficiency inhibited lipid accumulation in WAT (Figure 1G and 1H). No difference in food intake was determined, pointing that the reduced lipid accumulation was not due to energy intake (Figure 1I). Histomorphometric analysis revealed that the adipocytes from BAP31-ASKO mice were bigger than WT controls (Figure 1J). The analysis of adipocyte size frequency confirmed this observation, which showed increased number of hypertrophic adipocytes in BAP31-ASKO mice. The mean size of adipocytes in BAP31-ASKO mice is almost two folds than that of WT controls (Figure 1K), suggesting that BAP31-deficiency induced adipocyte expansion in mice. Liver and BAT weight were increased; no difference in skeletal muscles weight was observed (Table S3).

### BAP31-dificiency inhibited adipocyte differentiation

**Figure 2:**
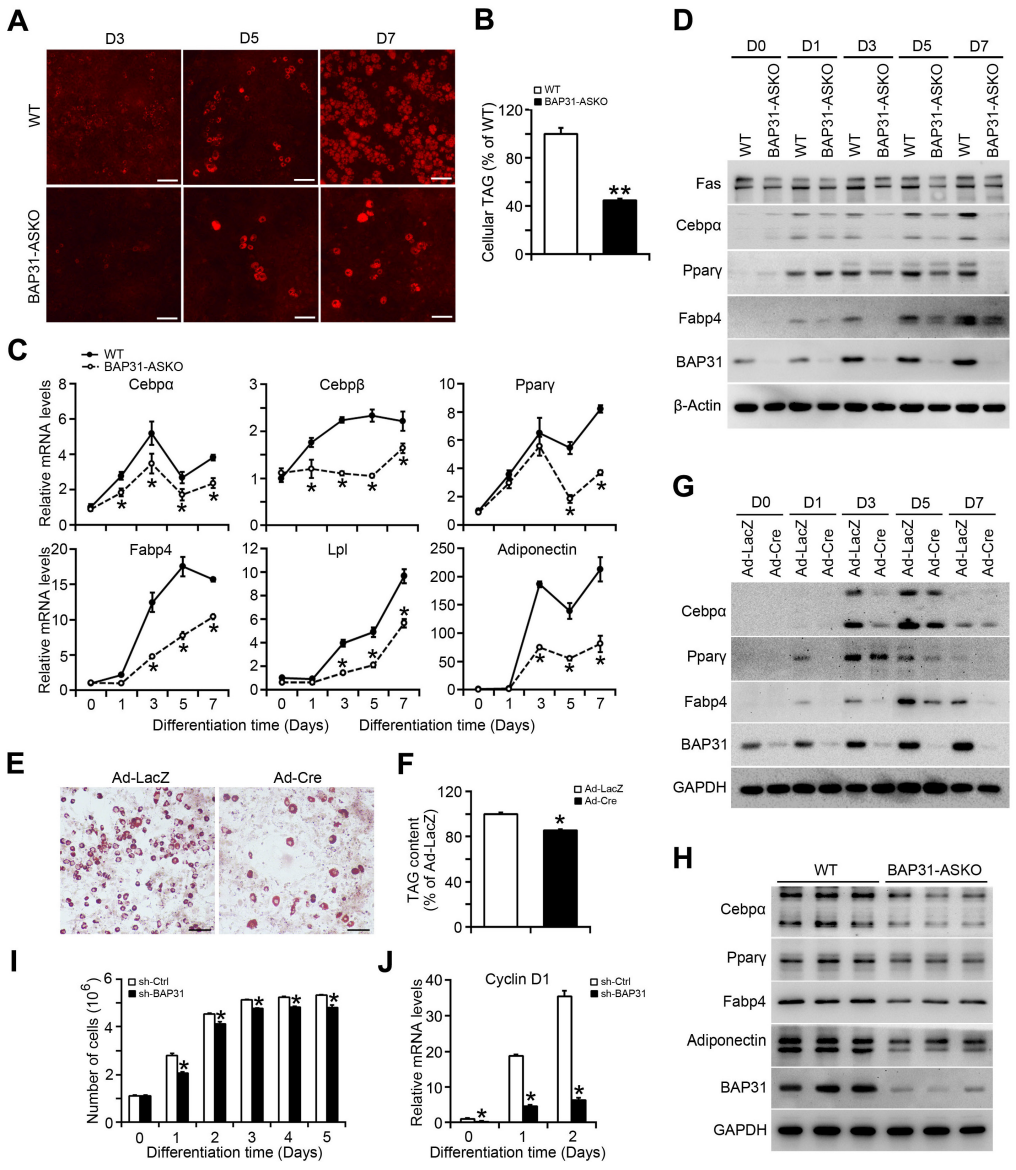
** BAP31-deficiency inhibited adipocyte differentiation. (A)** The representative images of Nile red staining of MEFs induced to differentiation to adipocytes. Scale bar=50 μm. **(B)** TAG content in differentiated MEFs. MEFs were isolated and induced to differentiation to adipocytes. The mature adipocytes were stained with Nile red at day 3 (D3), day 5 (D5), and day 7 (D7) post differentiation. Lipids were extracted from differentiated MEFs at day 7 post differentiation and TAG content was quantified. **p<0.01, compared to WT MEFs. **(C)** The transcriptional levels of adipogenic markers of Cebpα, Cebpβ, Pparγ, Fabp4, Lpl, and Adiponectin were reduced in BAP31-ASKO MEFs than WT controls. *p<0.05, compared to WT MEFs. **(D)** The protein levels of Fas, Cebpα, Pparγ, Fabp4, and BAP31 were determined in WT and BAP31-ASKO MEFs. **(E)** The representative images of oil red O staining of MEFs induced to differentiation to adipocytes. MEFs isolated from BAP31^flox/-^ or BAP31^flox/flox^ embryos were infected with adenovirus of Ad-LacZ and Ad-Cre to deplete BAP31 expression, and then were induced to differentiation to adipocytes. Cells were stained with oil red O at day 8 post differentiation. Scale bar=50 μm. **(F)** Lipids were extracted from differentiated MEFs and TAG content was measured. *p<0.05, compared to Ad-LacZ MEFs. **(G)** The protein levels of Cebpα, Pparγ, Fabp4, and BAP31 were determined in differentiated MEFs infected with Ad-LacZ and Ad-Cre adenovirus. **(H)** The protein levels of Cebpα, Pparγ, Fabp4, Adiponectin, and BAP31 were determined in WAT of WT and BAP31-ASKO mice. **(I)** BAP31-deficiency inhibited mitotic clonal expansion during 3T3-L1 preadipocytes differentiation induction process. 3T3-L1 preadipocytes differentiation was induced with the standard induction protocol. The cell number was determined at the indicated time after the induction to differentiation. *p<0.05, compared to sh-Ctrl.** (J)** The transcriptional levels of Cyclin D1 were determined in 3T3-L1 preadipocytes induced to differentiation to adipocytes. *p<0.05, compared to sh-Ctrl.

MEFs were isolated and induced to differentiation to adipocytes. Less staining of mature adipocytes was determined in BAP31-ASKO MEFs than WT controls (Figure 2A). Cellular TAG was reduced due to the reduced adipogenesis (Figure 2B). The transcriptional levels of the adipogenic markers, including CCAAT enhancer-binding protein alpha (Cebpα), Cebpβ, Peroxisome proliferator-activated receptor γ (Pparγ), Fatty acid binding protein 4 (Fabp4), Lipoprotein lipase (Lpl), and Adiponectin were decreased (Figure 2C). Immunoblotting analysis also reported decreased protein levels of Cebpα, Pparγ, Fabp4, and Fatty acid synthase (Fas) in BAP31-ASKO MEFs, demonstrating that BAP31-deficiency prevented MEFs differentiation to adipocytes (Figure 2D). In addition, BAP31^flox/-^ or BAP31^flox/flox^ MEFs infected with Ad-LacZ and Ad-Cre were induced to differentiation to adipocytes, which reported decreased mature adipocyte staining in Ad-Cre MEFs (Figure 2E). TAG content was decreased again (Figure 2F). The transcription and protein levels of adipogenic markers were both decreased in Ad-Cre MEFs (Figure 2G and Figure S1). Furthermore, the adipogenic markers in WAT were determined and displayed reduced expression pattern in BAP31-ASKO mice (Figure 2H), demonstrating that BAP31-deficiency inhibited the differentiation to adipocytes, and contributed to reducing lipid accumulation in WAT. After induction to differentiation to adipocytes, the cell numbers increased by 2-3 folds in control 3T3-L1 preadipocytes, but were reduced in sh-BAP31 cells, demonstrating that BAP31-deficiency inhibited the mitotic clonal expansion (MCE) and impaired the adipogenesis process (Figure 2I), which is in concordance with the reduced Cyclin D1 expression (Figure 2J).

### Lipid profiling

**Figure 3:**
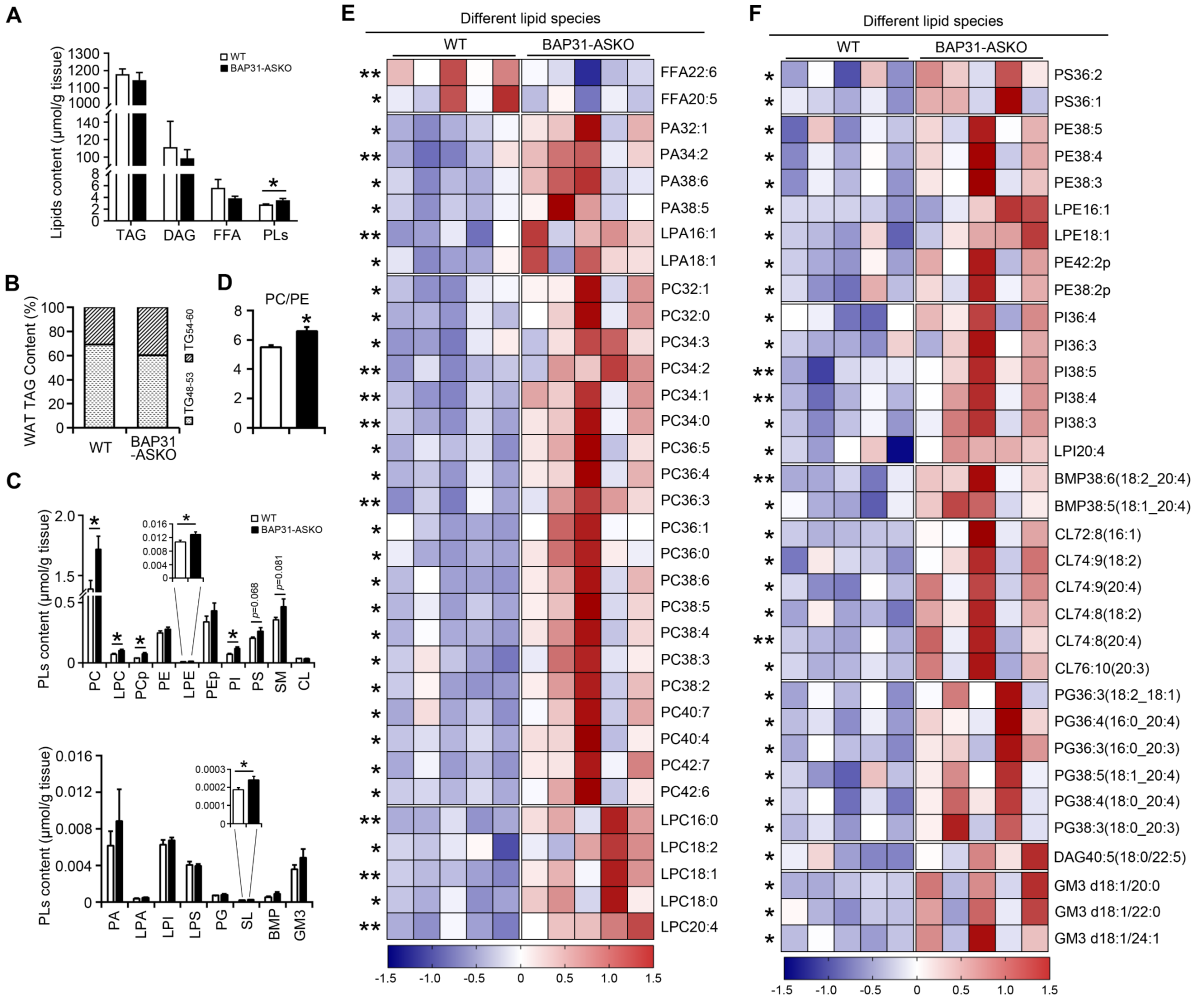
** The lipidomic analysis of epididymal white adipose tissue. (A)** The molar amount of TAG, DAG, FFA, and PLs was determined from epididymal white adipose tissue. **(B)** BAP31-deficiency promoted long chain PUFA-enriched TAG accumulation in white adipose tissue. **(C)** The species of PC, LPC, PCp, PE, LPE, PEp, PI, PS, SM, CL, and PA, LPA, LPI, LPS, PG, SL, BMP, GM3 were determined. **(D)** The ratio of PC/PE was increased in BAP31-ASKO mice. **(E and F)** The heatmap displaying statistically significantly regulated lipid molecular species between WT and BAP31-ASKO mice. *p<0.05, **p<0.01, compared to WT mice.

A total of 485 lipid species spanning 21 individual lipid classes was identified and quantitated in WAT lipidome. No significant difference in the total molar amount of TAG was determined (Figure 3A). However, TAG with carbon number of 48-53 was decreased, with carbon number of 54-60 was increased in BAP31-ASKO mice, suggesting that BAP31-deficiency modulated TAG ratio and facilitated long chain TAG accumulation in WAT (Figure 3B). No difference in DAG was determined (Figure 3A). Total amount of FFA was decreased insignificantly, with FFA22:6 and FFA20:5 were significantly decreased (Figure 3A and 3E). Total amount of phospholipids (PLs) was increased (Figure 3A), including the species of phosphatidylcholines (PC), lyso-PC (LPC), plasmalogen PC (PCp), plasmalogen phosphatidylethanolamines (LPE), phosphatidylinositols (PI), and sulfatides (SL) (Figure 3C). The PC/PE ratio was increased in BAP31-ASKO mice (Figure 3D). Figure 3E and 3F listed the significantly regulated lipid species between WT and BAP31-ASKO mice, suggesting that most PLs were up-regulated due to BAP31-deficiency.

### BAP31-deficiency reduced adipose tissue lipolysis

**Figure 4:**
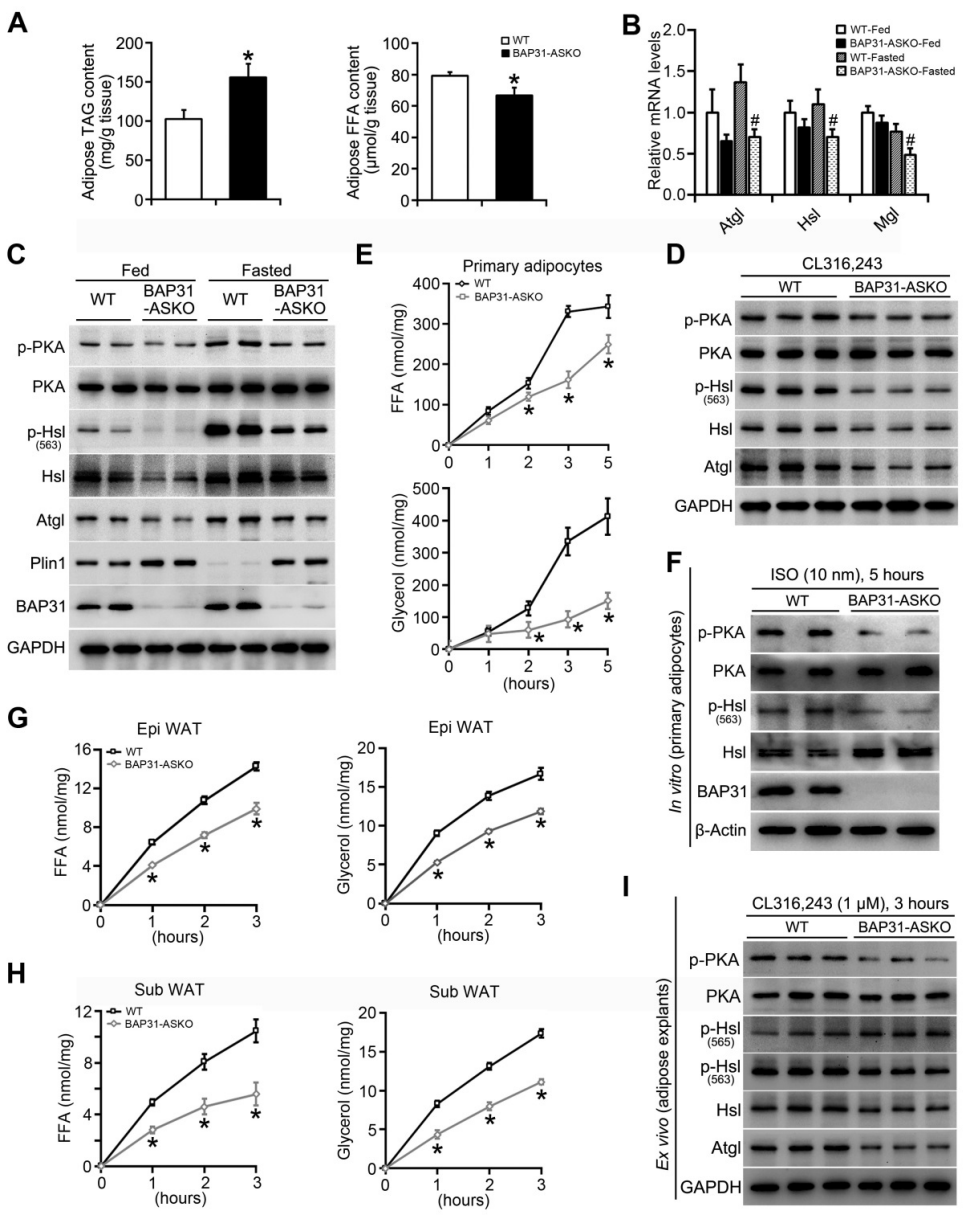
** BAP31-deficiency reduced adipose tissue lipolysis. (A)** TAG and FFA content in subcutaneous WAT of mice. n=6. *p<0.05, compared to WT mice. **(B and C)** BAP31-deficiency attenuated fasting response in mice. WT and BAP31-ASKO mice were food deprived for 24 hours. The epididymal WAT was dissected for future experiments. The mRNA levels of the lipase of Atgl, Hsl, and Mgl were reduced. n=6-7. ^#^*p*<0.05, compared to WT-Fasted mice (B). The protein levels of lipolysis-related genes of p-PKA, PKA, p-Hsl (563), Hsl, Atgl, Plin1, and BAP31 were determined (C). **(D)** BAP31-deficiency reduced β3-adrenoceptor agonist-induced lipolysis *in vivo*. WT and BAP31-ASKO mice were treated with CL316,243 (0.1 mg/kg), and then the epididymal WAT were dissected. The protein levels of p-PKA, PKA, p-Hsl (563), Hsl, Atgl, and BAP31 were determined. **(E and F)** BAP31-deficiency reduced ISO-induced lipolysis *in vitro*. The primary mature adipocytes were isolated and incubated with ISO (10 nM) for 5 hours. FFA and glycerol released into the medium were measured (E). The protein levels of p-PKA, PKA, p-Hsl (563), Hsl, and BAP31 were determined (F). **(G-I)** BAP31-deficiency reduced β3-adrenoceptor agonist-induced lipolysis *ex vivo*. The epididymal and subcutaneous WAT were dissected and minced into small pieces, followed by incubation with CL316,243 (1 μM) for 3 hours. FFA and glycerol released into the medium from epididymal (G) and subcutaneous WAT (H) were measured. The protein levels of p-PKA, PKA, p-Hsl (563), p-Hsl (565), Hsl, Atgl, and BAP31 were determined in white adipose explants (I). *p<0.05, compared to WT mice.

TAG in WAT was increased in BAP31-ASKO mice, accompanied by decreased FFA content (Figure 4A). We suggested that BAP31-deficiency reduced adipose tissue lipolysis, which leaded to increased TAG accumulation and decreased FFA release in adipocytes. Thus, mice were food deprived and the lipolytic effects were determined. BAP31-deficiency blocked fasting-induced hypoglycemia, reduced TAG content, and repressed serum FFA and glycerol releasing from WAT via lipolysis. No difference in cholesterol was determined (Table 1). The mRNA levels of Adipose triglyceride lipase (Atgl), Hsl, and Monoglyceride lipase (Mgl) were reduced in BAP31-ASKO-Fasted mice (Figure 4B), as well as PKA signaling activation, p-Hsl (563) phosphorylation and Atgl expression, pointing that BAP31-deficiency attenuated fasting-induced lipolysis (Figure 4C). This attenuation was not observed in liver tissues, demonstrating that the reduced lipolysis is due to the specific depletion of BAP31 in adipocytes (Figure S2). It was noted that BAP31-deficiency increased Plin1 expression at fed and fasted status (Figure 4C). Again, mice were treated with β3-adrenergic receptor agonist. BAP31-deficiency increased blood glucose, but decreased FFA and glycerol release from WAT. No difference in TAG was determined (Table 2). Consistently, p-PKA and p-Hsl (563) were reduced, along with reduced Atgl expression, suggesting that BAP31-deficiency reduced CL316,243-induced lipolysis in mice (Figure 4D).

Next, the lipolytic effects were explored from primary adipocytes. The release of FFA and glycerol increased along with ISO-treatment, but was attenuated in BAP31-ASKO adipocytes (Figure 4E). ISO-induced PKA signaling activation was reduced, accompanied by reduced p-Hsl (563) levels, demonstrating that BAP31-deficiency inhibited ISO-induced lipolysis* in vitro* (Figure 4F). Furthermore, *ex vivo* lipolysis was performed on epididymal and subcutaneous WAT explants. CL316,243 increased FFA and glycerol release, but the induction was repressed in BAP31-ASKO explants (Figure 4G and 4H). PKA signaling activation was reduced, as well as p-Hsl (563) levels. It was noted that p-Hsl (565) levels, which were phosphorylated by AMPK signaling 27, exhibiting no difference between WT and BAP31-ASKO explants, suggesting that AMPK signaling may not be involved in BAP31 function on lipolysis (Figure 4I). BAP31-deficiency in adipocytes reduced the total lipase activity in epididymal WAT, as well as in quadriceps muscles, but not in serum and liver tissues (Figure S3).

### BAP31-deficiency caused lipid droplets abnormal growth

**Figure 5:**
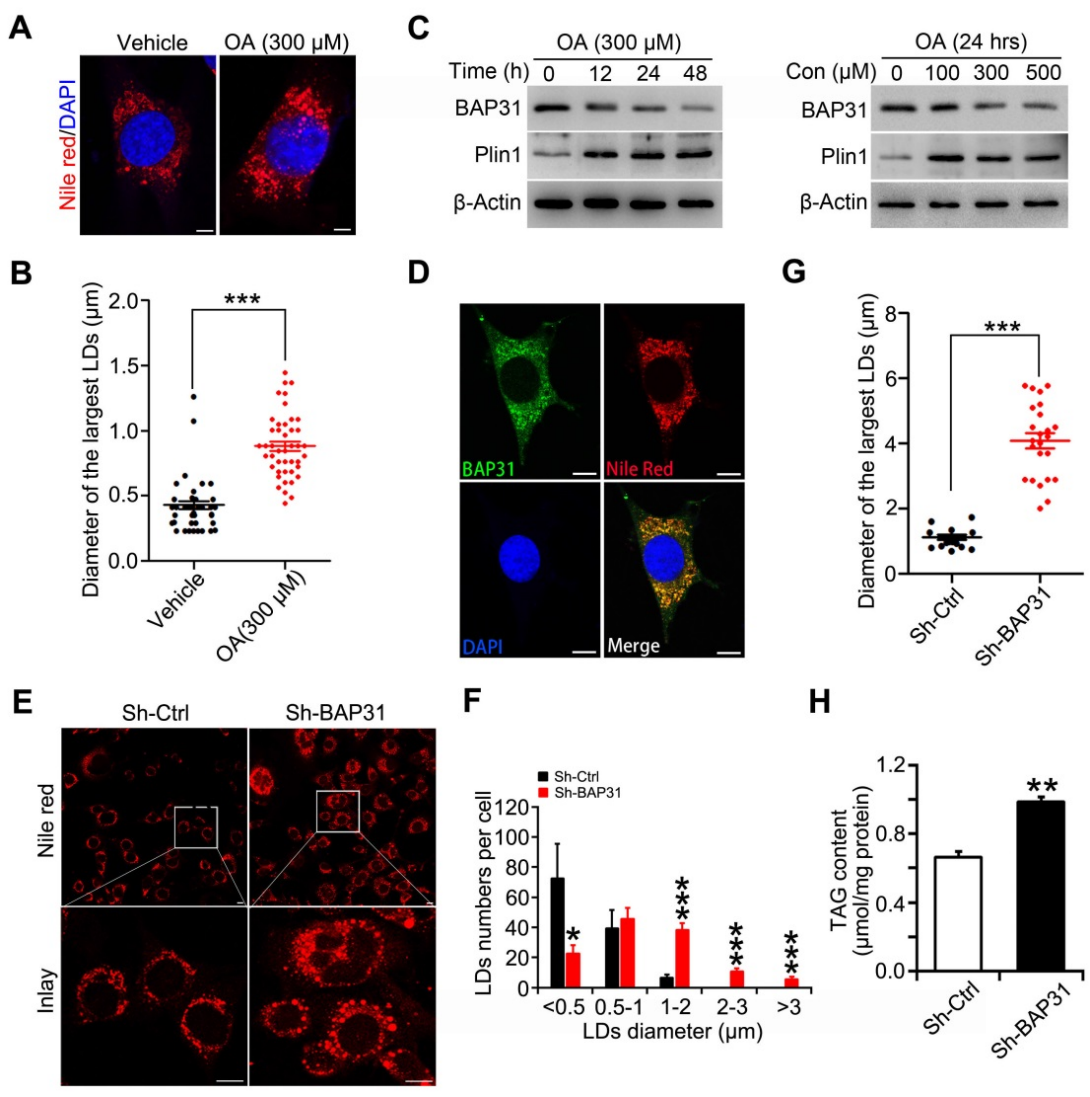
** BAP31-deficiency caused lipid droplets abnormal growth. (A and B)** Oleic acid increased LD size in 3T3-L1 preadipocytes. 3T3-L1 preadipocytes were treated with oleic acid (300 μM) for 48 hours. LDs were visualized via Nile red staining (A). The diameter of the largest LDs was analyzed (B). ***p<0.001, compared to vehicle control. **(C)** Oleic acid reduced BAP31 protein levels and induced Plin1 protein levels in 3T3-L1 preadipocytes. 3T3-L1 preadipocytes were treated with 300 μM of oleic acid for 0, 12, 24, 48 hours, or with 0, 100, 300, 500 μM of oleic acid for 24 hours. **(D)** BAP31 colocalizes with LDs in 3T3-L1 preadipocytes. 3T3-L1 preadipocytes cultured on coverslips were treated with oleic acid (300 μM, 48 hours), then stained with anti-BAP31 antibody (green), Nile red for lipid droplets (red), and DAPI for nucleus (blue). Scale bar=15 μm. **(E-H)** BAP31-deficiency increased LD size and lipid accumulation in 3T3-L1 preadipocytes. The stable cell line of BAP31-deficiency (sh-BAP31) and control (sh-Ctrl) were treated with 300 μM of oleic acid for 48 hours, then stained with Nile red for LDs and visualized by confocal microscope. BAP31-deficiency alters the morphology in 3T3-L1 preadipocytes. Scale bar=15 μm (E). Histogram showing the mean number of LDs per cell in each diameter in E (F). BAP31-deficiency increased the diameter of the largest LDs in 3T3-L1 preadipocytes (G). BAP31-deficiency increased TAG content in 3T3-L1 preadipocytes (H). *p<0.05, ***p<0.001, compared to Sh-Ctrl.

BAP31 is one of the integral ER membrane proteins and LDs are ER-derived neutral lipid storage organelles. We suggested that BAP31 localizes on LDs surface via ER budding, and regulates TAG hydrolysis and LD size. Oleic acid (OA) increased lipid accumulation in 3T3-L1 preadipocytes and induced LDs enlargement (Figure 5A and 5B). In accompaniment with OA treatment, Plin1 was increased significantly in 3T3-L1 preadipocytes. On the contrary, BAP31 was reduced via a time- and dosage-dependent pattern, exhibiting a negative correlation with Plin1 expression (Figure 5C). BAP31 colocalizes with LDs, pointing the possible roles in LDs growth (Figure 5D). 3T3-L1 preadipocytes depleted with BAP31 were induced with OA and LD size was determined. The results demonstrated that BAP31-deficiency increased LD size and promoted LDs abnormal growth (Figure 5E). In control preadipocytes, a large number of small LDs accumulated in the presence of OA. However, in BAP31-deficient preadipocytes, reduced a few small LDs were observed, and these LDs expanded rapidly grew into supersized ones (Figure 5F and 5G). Cellular TAG was increased in BAP31-deficient preadipocytes, attributing to the reduced TAG hydrolysis (Figure 5H).

### BAP31 increased lipolysis and rescued lipid droplets abnormal growth via modulating Perilipin1 expression

**Figure 6:**
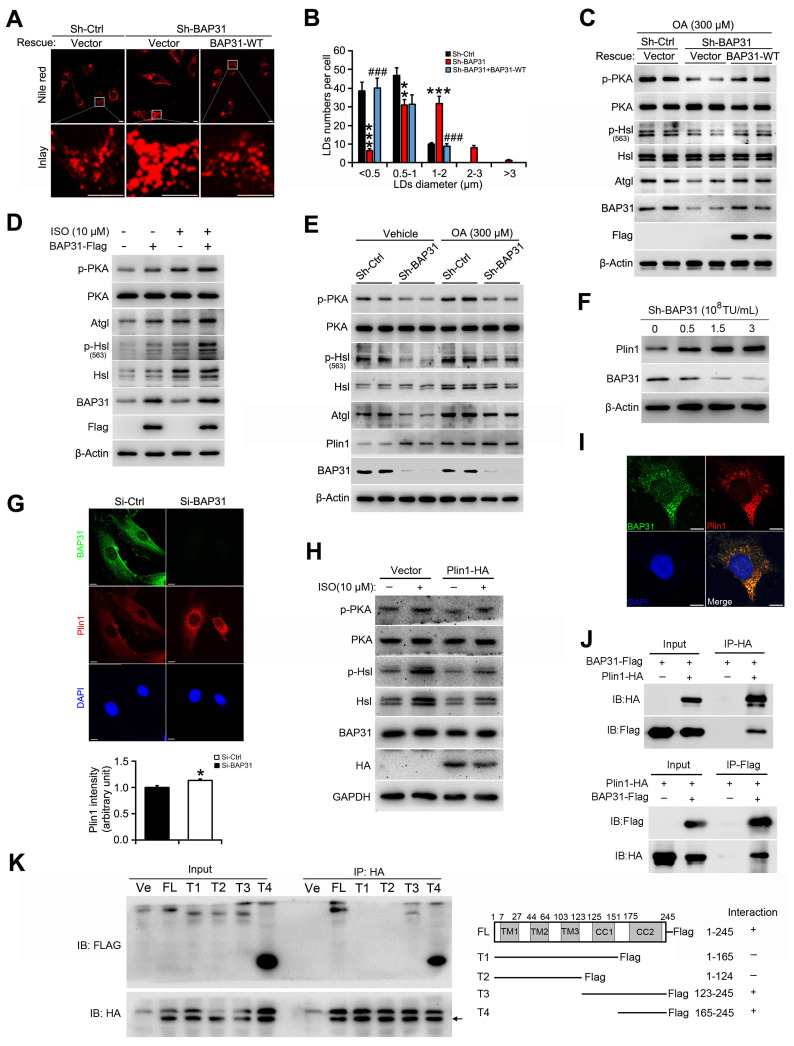
** BAP31 increased lipolysis and rescued lipid droplets abnormal growth via modulating Perilipin1 expression. (A)** Over-expression of BAP31 rescued the induction of LD expansion due to BAP31-deficiency. **(B)** Histogram showing the mean LDs number in each diameter. **p<0.01 and ***p<0.001, compared to sh-Ctrl. ^###^*p*<0.001 compared to sh-BAP31. **(C)** The protein levels of lipolysis-related genes of p-PKA, PKA, p-Hsl (563), Hsl, and Atgl were determined. **(D)** Over-expression of BAP31 increased ISO-induced lipolysis. 3T3-L1 preadipocytes transfected with BAP31-Flag were incubated with ISO (10 μM) for 24 hours, then the protein levels of p-PKA, PKA, p-Hsl (563), Hsl, and Atgl were determined. **(E)** BAP31-deficiency promoted Plin1 protein levels in oleic acid-treated 3T3-L1 preadipocytes. **(F)** Reduced BAP31 expression resulted in enhanced Plin1 protein levels. 3T3-L1 preadipocytes were infected with different titer of lentivirus targeted with BAP31 for 72 hours. The protein levels of BAP31 and Plin1 were determined then. **(G)** BAP31-deficiency increased Plin1 fluorescence intensity. 3T3-L1 preadipocytes cultured on coverslips were transfected with si-Ctrl and si-BAP31 for 72 hours, and then were fixed and immunostained with anti-BAP31 (green), anti-Plin1 (red), and DAPI (blue). The relative fluorescence intensity was calculated. Scale bar=10 μm. *p<0.05 compared to Si-Ctrl. **(H)** Enforced expression of Plin1 repressed ISO-induced PKA signaling activation. 3T3-L1 preadipocytes transfected with Plin1-HA or vector were incubated with ISO (10 μM) for 4 hours. The cells were lysed with RIPA buffer and the protein levels of p-PKA, PKA, p-Hsl (563), Hsl, and Atgl were determined. **(I)** BAP31 colocalizes with Plin1 in 3T3-L1 preadipocytes. 3T3-L1 preadipocytes cultured on coverslips were immunostained by anti-BAP31 (green), anti-Plin1 (red), and DAPI (blue). Scale bar=10 μm. **(J)** Co-IP demonstrated the interaction of BAP31 and Plin1 in 3T3-L1 preadipocytes. **(K)** Mapping interaction domains in BAP31 and Plin1. A series of BAP31 mutants with Flag-tag (T1, T2, T3, and T4) were co-transfected with Plin1-HA in 3T3-L1 preadipocytes. Protein extracts were immunoprecipitated with an anti-HA antibody. Immunoprecipitants were analyzed by immunoblotting analysis with an anti-Flag antibody. FL: full length. Ve: vector.

When BAP31 was reintroduced into BAP31-deficient preadipocytes, LD size was decreased and the number of visible LDs were decreased, showing a restoration of normal LD morphology (Figure 6A and 6B). In accordance with the morphological observation, the reduced PKA signaling activation was restored. Increased p-PKA and p-Hsl phosphorylation levels, and increased Atgl expression were determined due to enhanced BAP31 expression, promoted ISO-induced TAG hydrolysis, thus modulated the supersized LDs to normal morphology (Figure 6C and 6D). OA increased Plin1 protein stability and/or protein levels, stabilized LD dynamics and promoted the formation of supersized LDs (Figure 6E). BAP31-deficiency increased Plin1 protein levels (Figure 4B and 6E). Whether BAP31 regulates LD size via modulating Plin1 protein levels and/or protein stability in adipocytes? Gene silencing reduced BAP31 expression dosage-dependently, which induced Plin1 expression and exhibited a negative correlation consequently (Figure 6F and 5C). Immunofluorescence assay confirmed this observation, displaying enhanced Plin1 staining due to a transient deficiency of BAP31 expression (Figure 6G). Enhanced Plin1 expression repressed ISO-induced PKA signaling activation and reduced the lipolysis, illustrating the possibility of that BAP31 regulated LDs growth and LDs degradation via modulating Plin1 protein (Figure 6H). Thus, the interaction of BAP31 and Plin1 were determined. BAP31 colocalizes with Plin1 on LDs surface (Figure 6I). Co-IP assay further demonstrated that BAP31 and Plin1 interacts with each other (Figure 6J). A series of BAP31 mutants with Flag-tag were exogenously expressed together with HA-tagged Plin1 in 3T3-L1 preadipocytes. Cell lysates were then immunoprecipitated using an antibody against HA and analyzed by immunoblotting analysis. The truncated BAP31 containing amino acids 123-245 (123-245-Flag) and 165-245 (165-245-Flag) interacted with Plin1, whereas the mutants which lack the C-terminal cytoplasmic portion of the protein (1-165-Flag and 1-124-Flag) completed abolished the interaction (Figure 6K), suggested that amino acids 165-245 of BAP31 are necessary for its interaction with Plin1.

### BAP31 regulated Perilipin1 expression via modulating the proteasomal degradation

**Figure 7:**
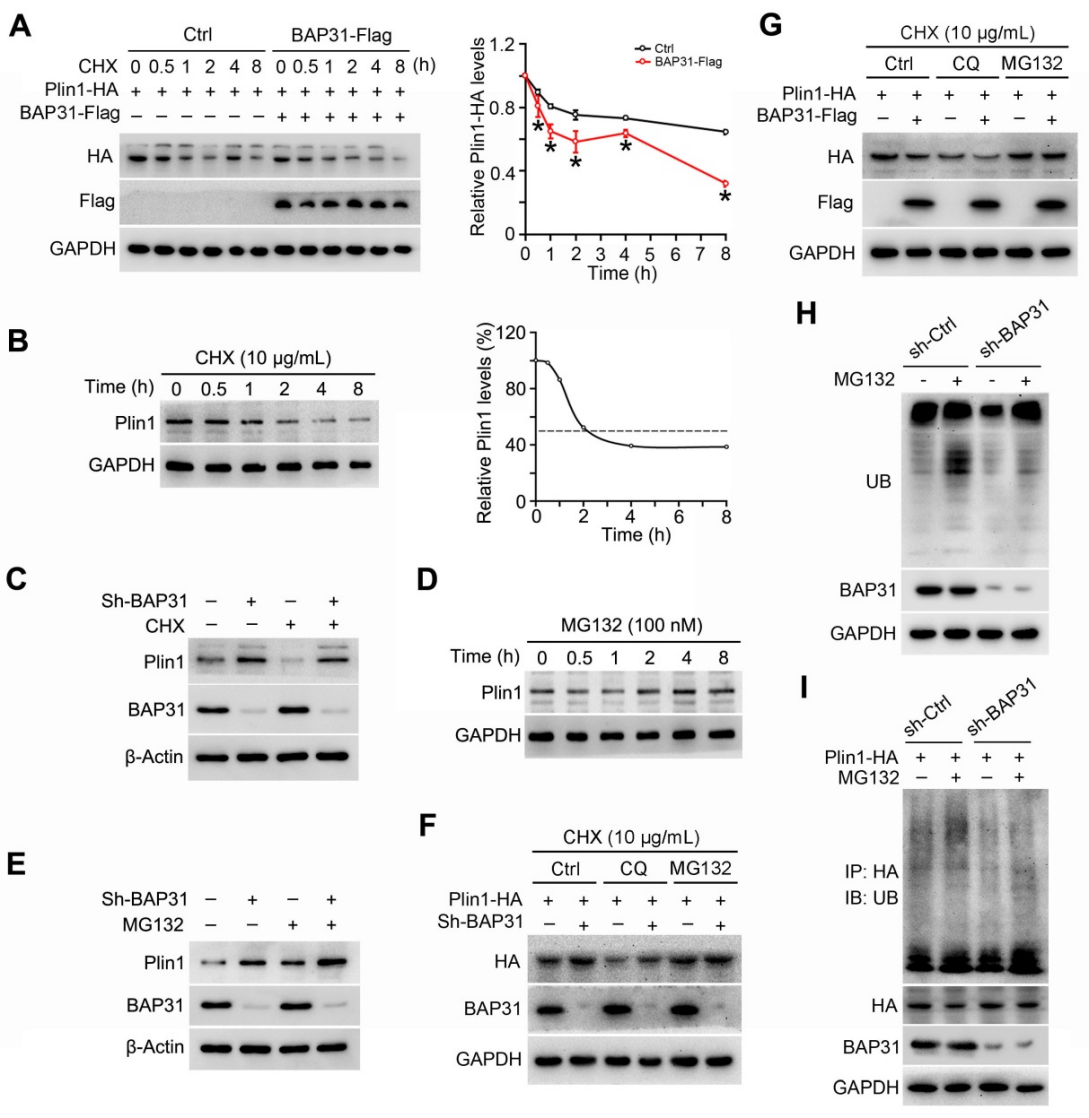
** BAP31 regulated Perilipin1 expression via modulating the proteasomal degradation. (A)** BAP31 promoted Plin1-HA degradation. 3T3-L1 preadipocytes transfected with Plin1-HA and BAP31-Flag plasmids were treated with CHX (10 μg/mL) for 0, 0.5, 1, 2, 4, and 8 hours. Plin1-HA and BAP31-Flag expression were determined using immunoblotting analysis. The degradation curve was calculated based on the protein quantification of Plin1-HA. *p<0.05, compared to Ctrl group. **(B)** 3T3-L1 preadipocytes were treated with CHX (10 μg/mL) for 0, 0.5, 1, 2, 4, and 8 hours, and then Plin1 protein levels were determined. **(C)** BAP31-deficiency increased Plin1 protein levels in CHX-treated preadipocytes. **(D)** 3T3-L1 preadipocytes were treated with 100 nM of MG132 for 0, 0.5, 1, 2, 4, and 8 hours, and then Plin1 protein levels were determined. **(E)** BAP31-deficiency promoted Plin1 protein levels with or without MG132 treatment. **(F)** The effects of BAP31-deficiency increasing Plin1-HA expression were prevented by proteasomal inhibition, not via lysosomal inhibition. Sh-Ctrl and sh-BAP31 3T3-L1 preadipocytes were transfected with Plin1-HA plasmids, and then were treated with CHX (10 μg/mL) for 2 hours. After that, cells were treated with chloroquine (CQ, 15 mM) or MG132 (100 nM) for another 2 hours. Immunoblotting analysis was performed with the cell lysates. **(G)** BAP31 reduced Plin1-HA expression was prevented via proteasomal inhibition, not via lysosomal inhibition. 3T3-L1 preadipocytes were transfected with BAP31-Flag and Plin1-HA plasmids, and then were treated with CHX (10 μg/mL) for 2 hours. After that, cells were treated with chloroquine (15 mM) or MG132 (100 nM) for another 2 hours. **(H)** BAP31 is needed for the proteasomal degradation. Sh-Ctrl and sh-BAP31 3T3-L1 preadipocytes were treated with MG132 (100 nM) for 2 hours. The ubiquitinated protein was detected using an anti-Ub antibody. **(I)** BAP31-deficiency repressed Plin1 proteasomal degradation in 3T3-L1 preadipocytes. Sh-Ctrl and sh-BAP31 3T3-L1 preadipocytes were transfected with Plin1-HA plasmids, and then treated with MG132 (100 nM) for 2 hours. Cell lysates were immunoprecipitated with an anti-HA antibody. The ubiquitinated Plin1-HA was detected via immunoblotting analysis.

3T3-L1 preadipocytes were transfected with Plin1-HA and BAP31-Flag plasmids, and then were treated with the protein synthesis inhibitor of cycloheximide (CHX), demonstrating that BAP31 leaded to the instability of Plin1 protein (Figure 7A). Therefore, cells were treated with CHX in a time-course experiment. Plin1 protein levels were decreased and reached to ~50% at 2 hours after CHX treatment, implying protein degradation involved in Plin1 turnover (Figure 7B). BAP31 was depleted in 3T3-L1 preadipocytes, which induced Plin1 expression and exhibited even higher levels than that with the presence of CHX, suggesting that BAP31-deficiency inhibited the protein degradation of Plin1 (Figure 7C). MG132, the proteasomal inhibitor, time-dependently increased Plin1 expression (Figure 7D). BAP31-deficiency enhanced the increase, pointing the possibility of that knockdown of BAP31 attenuated the proteasomal degradation of Plin1 (Figure 7E). To confirm the proteasomal protein degradation involved in Plin1 turnover, 3T3-L1 preadipocytes were transfected with Plin1-HA, and then were treated with CHX, followed by incubation with the lysosomal inhibitor of chloroquine (CQ) or MG132. The results demonstrated that MG132, instead of CQ, blocked BAP31-deificiency induced Plin1 increase (Figure 7F). In contrast, BAP31 reduced Plin1 expression, MG132 not CQ repressed the reduction of Plin1 expression (Figure 7G), suggesting that the proteasomal degradation, not the lysosomal degradation involved in BAP31-mediated Plin1 turnover. MG132 inhibits the proteolytic activity of the 26S proteasome complex, results in the accumulation of ubiquitin-conjugated proteins, but was reduced in BAP31-deficient preadipocytes, illustrating that BAP31 is needed and essential for the proteolytic activity of the proteasome. (Figure 7H). Co-IP assay further demonstrated that MG132 increased the protein levels of ubiquitinated Plin1-HA, but was totally blocked in sh-BAP31 preadipocytes, suggesting that BAP31-deficiency inhibited Plin1 ubiquitination and decreased the proteasomal degradation (Figure 7I).

### BAP31-deficiency reduced HFD-induced obesity, but attenuated insulin signaling and increased the inflammatory response in mice

**Figure 8:**
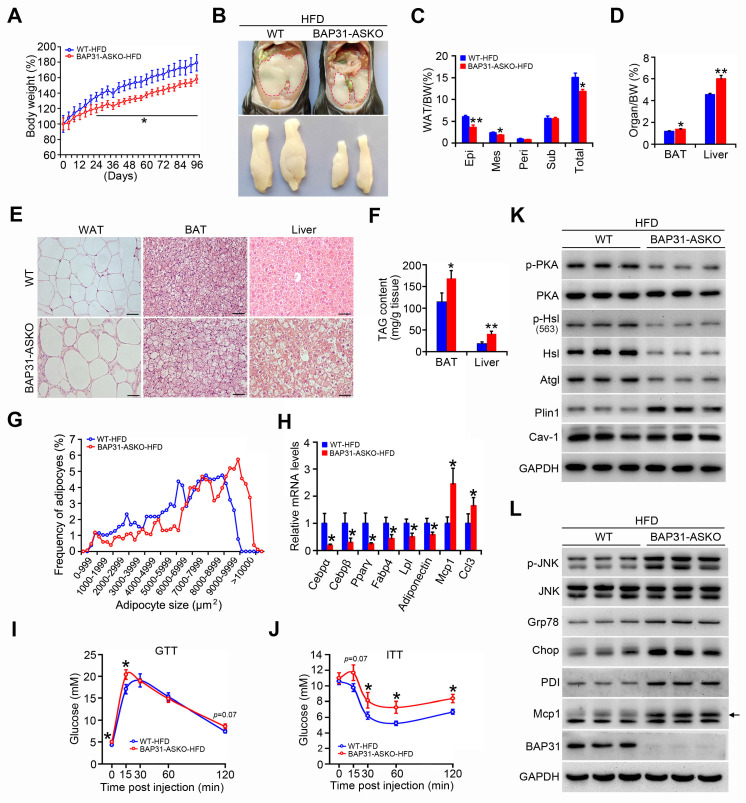
** BAP31-deficiency reduced HFD-induced obesity, but attenuated insulin signaling and increased the inflammatory response in mice. (A)** BW change was reduced in BAP31-ASKO mice upon HFD-feeding. **(B)** The representative images of epididymal WAT. **(C)** The epididymal WAT (Epi), mesenteric WAT (Mes), perirenal WAT (Peri), subcutaneous WAT (Sub), and total WAT mass were recorded in WT and BAP31-ASKO mice with HFD-feeding. **(D)** The organ indexes of BAT and liver. **(E)** The representative images of H/E staining of epididymal WAT, BAT, and liver. Scale bar=50 μm. n=4. **(F)** TAG content of BAT and liver. **(G)**The frequency of adipocytes in epididymal WAT from (E). **(H)** The mRNA levels of Cebpα, Cebpβ, Pparγ, Fabp4, Lpl, Adiponectin, Mcp1, and Ccl3 were determined. **(I and J)** Glucose and insulin tolerance tests were performed with WT and BAP31-ASKO mice. **(K and L)** The protein levels of p-PKA, PKA, p-Hsl (563), Hsl, Atgl, Plin1, Cav-1, and p-JNK, JNK, Grp78, Chop, PDI, Mcp1 were determined. *p<0.05, **p<0.01, compared to WT-HFD mice.

Upon HFD-feeding, BAP31-ASKO mice exhibited reduced BW increase than WT controls (Figure 8A). The epididymal pads were smaller, along with reduced Epi, Mes and total WAT mass, suggesting that BAP31-deficiency prevented diet-induced lipid accumulation in WAT (Figure 8B and 8C). More bigger adipocytes were observed in BAP31-ASKO mice. The analysis of adipocyte size confirmed this observation, demonstrating that BAP31-deficiency promoted adipocyte expansion, keeping consistency with the results from chow diet (Figure 8E and 8G). BAT and liver weight were increased in BAP31-ASKO mice (Figure 8D), accompanied by enhanced ectopic lipid accumulation (Figure 8E and 8F). BAP31-deficiency reduced the adipogenic markers of Cebpα, Cebpβ, Pparγ, Fabp4, Lpl, Adiponectin expression, and induced Monocyte chemotactic protein-1 (Mcp1) and C-C motif chemokine ligand 3 (Ccl3) expression (Figure 8H). BAP31-ASKO mice exhibited higher glucose at 0 and 15 minutes, and insignificant higher at 120 minutes (*p*=0.07) upon glucose challenge (Figure 8I). For the ITT assay, enhanced glucose was determined at 30, 60, and 120 minutes, with insignificant increase at 15 minutes (*p*=0.07) (Figure 8J), demonstrating that BAP31-deficiency in adipocytes reduced insulin signaling in mice. The phosphorylation levels of p-PKA and p-Hsl were decreased, along with decreased Hsl and Atgl protein levels in WAT of BAP31-ASKO mice upon HFD-feeding. Plin1 was increased, and no difference of Caveolin-1 (Cav-1) was determined (Figure 8K), pointing that BAP31 effects on Plin1 protein regulation are specific. The protein levels of Glucose-regulated protein 78 (Grp78), C/EBP homologous protein (Chop), Protein disulfide isomerase (PDI), and p-JNK, Mcp1 were increased in BAP31-ASKO mice, suggesting enhanced ER stress and promoted pro-inflammatory response in WAT (Figure 8L).

## Discussion

**Figure 9:**
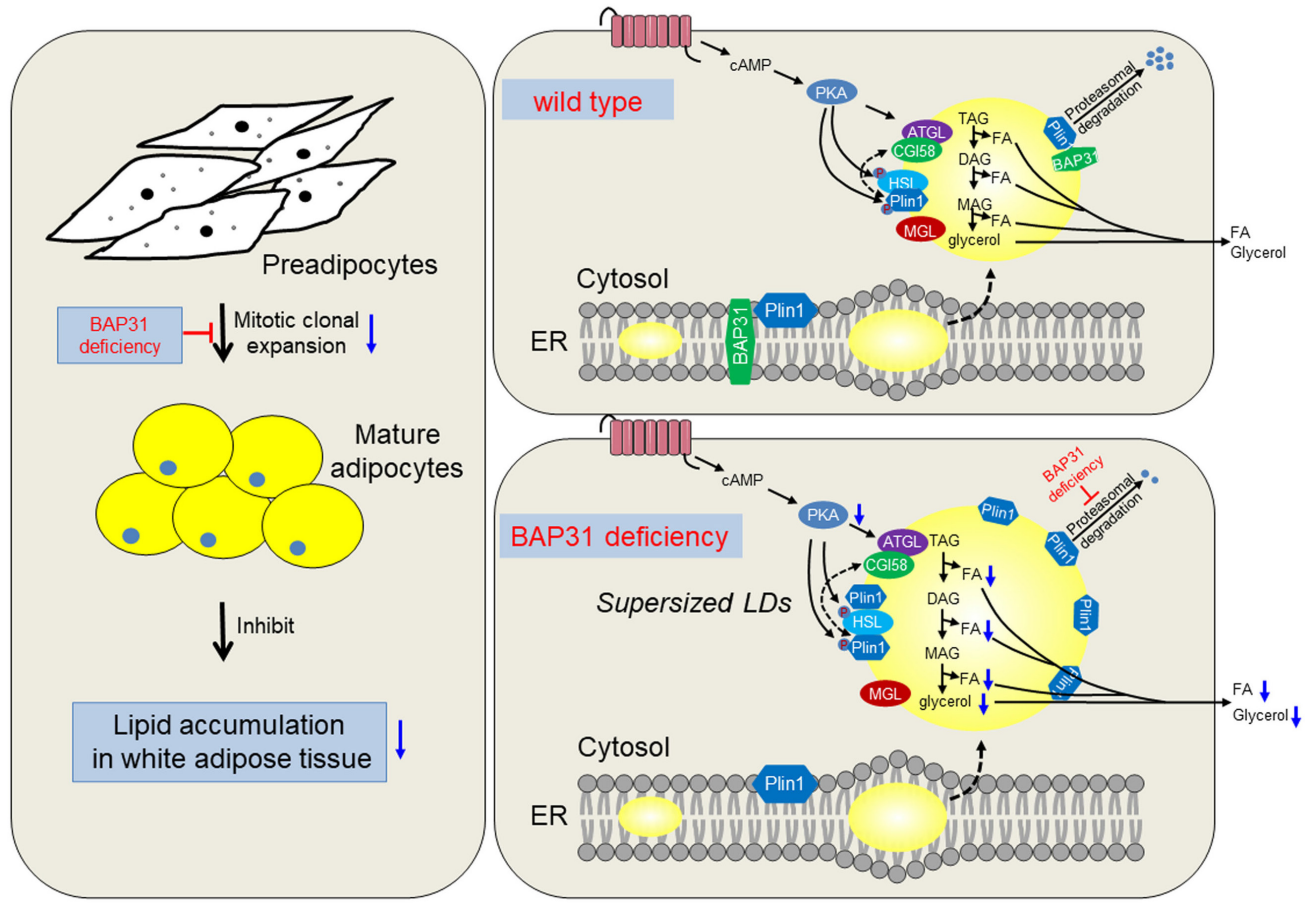
** The working model of BAP31 function on lipid metabolism in adipocytes.** BAP31-deficiency reduced mitotic clonal expansion, attenuated adipogenesis and lipid accumulation in white adipose tissue; expanded adipocytes size and promoted LDs abnormal growth through attenuating LDs hydrolysis via preventing Perilipin1 proteasomal degradation in mice.

Currently we reported the novel roles of BAP31 in regulating lipid metabolism in adipocytes. BAP31-deficiency inhibited MCE, reduced adipogenesis, and prevented lipid accumulation in WAT. Also, BAP31 collaborates with Plin1 by the C-terminal cytoplasmic portion and regulates Plin1 protein levels via modulating the proteasomal degradation. BAP31-deficiency blocked Plin1 degradation and increased Plin1 expression on LDs surface, attenuated LDs degradation and promoted the formation of supersized LDs. Furtherly, BAP31-deficiency inhibited PKA-signaling activation and the lipolysis process, resulted in adipocyte expansion, which promoted inflammation in WAT and impaired insulin signaling in mice (Summary in Figure 9). Based on our knowledge, this is the first report illustrating BAP31 function in regulating adipocyte differentiation, as well as LDs biogenesis and degradation in adipocytes, maintaining LDs homeostasis via modulating the LD-associated protein proteasomal degradation.

BAP31-deficiency promoted Plin1 protein levels on LDs surface, and played the protective role against agonist-induced lipolysis. The current study reported the novel roles of BAP31 in regulating LD-associated protein degradation and modulating LDs growth, strengthening the biological function of BAP31 in lipid metabolism in adipocytes. BAP31 locates on LDs surface and regulated lipid metabolism via LD catabolism, serving as one of LD-associated proteins in adipocytes 11. BAP31 accumulates at the juxtanuclear region of a few cells, interacted with Tom40 and formed the mitochondrial complex I, facilitated the translocation of NDUFS4, representing a mechanism for the ER-mitochondria communication 28. We suggested that BAP31 may undergo regulated translocation from the ER to the LDs, raising the theoretical possibility that traffic between the phospholipid monolayer of the LDs and the ER. Lipolysis is mediated by the activation of a PKA-mediated pathway, promotes TAG hydrolysis on LDs surface by the sequential activation of Atgl, Hsl, and Mgl lipases 29. BAP31-deficiency reduced PKA signaling activation, decreased Atgl expression, accompanied by reduced Hsl phosphorylation, eventually reduced the lipolysis rate in adipocytes (Figure 4). Reduced lipolysis leaded to enhanced lipid accumulation and TAG content in adipocytes, resulted in the formation of supersized LDs, which is in agreement with the observation of increased TAG content in OA-induced sh-BAP31 preadipocytes (Figure 5H), also keeping consistency with the previous study of that BAP31-deficiency in hepatocytes promoted hepatic lipid accumulation and worsened insulin resistance 21. LDs are emerging as dynamic cellular organelles and play a crucial role in lipid and membrane homeostasis. LDs size and protein compositions vary between cell type and the underlying conditions. Upon lipid overloading, cells can respond to lipids storage pressure by either increasing the number and/or the volume of the LDs. Supersized LDs provided the most efficient ways for fat storage that is necessary and beneficial under lipid overloading or lipotoxic conditions. Enhanced Plin1 leaded to the formation of large unilocular LDs that are a unique of WAT. Nascent LDs in adipocytes are coated with Plin3 and Plin4, and replaced by Plin1 on the surface of giant and unilocular LDs, which facilitates the formation of giant LDs 30, suggesting the possibility of the formation of supersized LDs with enhanced Plin1 expression in BAP31 deficient adipocytes.

BAP31 was reported playing an integral role in the recognition of misfolded protein by triggering ER-associated degradation, and considered as a component of the ER quality control compartment 31, recognized the newly synthesized CFTRΔF508 and promoted the retro-translocation from the ER and the degradation by the 26S proteasome system 16, pointing the key roles of BAP31 in regulating protein stability and the related protein degradation. Two independent groups have proposed different domains of BAP31 to be critical for the interaction. Annaert* et al.* demonstrated that the three transmembrane regions of BAP31 are required for the binding with cellubrevin 32. Ducret *et al.* reported that the cytoplasmic domain of BAP31 is responsible for the interaction with γ-actin 33. BAP31 translocates from the ER to the LDs, and interacts with Plin1 on the LDs surface. Immunoprecipitation assay demonstrated that the C-terminal cytoplasmic portion is essential and critical for the interaction (Figure 6). This specific interaction of Plin1 and BAP31 is supported by the presence of a coiled-coil region of BAP31, in which α-helices intertwine to form a superhelical bundles and has been considered as the simplest of all protein interaction motifs 34, and may facilitate the controlling of Plin1 turnover via the proteasomal protein degradation. Plin1 protein levels on LDs surface are dynamically regulated, via modulating the ubiquitination-proteasome pathway 9. Given the roles of Plin1 in protecting LDs lipids from lipase hydrolysis, it is reasonable to anticipate that the assembly of Plin1 in macrophages might reduce lipolysis and hence increase lipid retention in ApoE-deficiency plaques 35. Reduced BAP31 expression repressed the proteasomal degradation of Plin1 and leaded to Plin1 accumulation on LDs surface, blocked agonist-induced PKA signaling activation and the lipolysis in adipocytes. This study shows the essential roles of BAP31 in controlling the homeostasis of LD-associated surface proteins via the proteasomal degradation system, suggesting the important function of BAP31 in regulating protein quality control and LD metabolism.

BAP31-deficiency reduced adipogenesis and lipid accumulation in WAT, even though the induced adipocytes expansion and enhanced cellular TAG accumulation due to reduced TAG hydrolysis on LDs surface, suggesting that the effects of reduced adipogenesis with reduced lipid accumulation in WAT overwhelmed the consequence of increased TAG accumulation due to reduced lipolysis rate within adipocytes. Two *in vitro* MEFs cell culture models demonstrated that BAP31-deficiency repressed the expression of adipogenic markers, and prevented the differentiation to adipocytes. When induced to differentiation, growth arrested preadipocytes synchronously reenter the cell cycle and undergo the required process of MCE, followed by the expression of genes aiming to adipocyte phenotype, including Cyclin D1, Cebpβ, cdk2-cyclin E, and so on 36, 37. BAP31-deficiency inhibited Cyclin D1 expression and reduced MCE in 3T3-L1 preadipocytes, consequently attenuated the process of adipogenesis. cAMP-PKA signaling is important both in adipogenesis and lipolysis in WAT, and cAMP-dependent PKA activation promotes adipogenesis 38. PKA signaling was decreased after BAP31 depletion, contributed to reducing adipocyte differentiation and lipid accumulation in WAT of BAP31-ASKO mice. Reduced PKA signaling activation decreased TAG hydrolysis and reduced FAs release from the adipocytes. The reduced FAs content furtherly repressed Pparγ signaling and inhibited the adipogenesis process 39. We failed to detect the direct evidence of BAP31 regulation on Cebpα and Pparγ transcription. Whether BAP31 traffics into the nuclear or fuses with the nuclear membrane via LDs transportation or fusion, and then regulates the adipogenic marker transcription by direct binding to the promoter element will be interested in future studies 3, 40. Reduced adipogenesis prevented excessive lipids incorporating into WAT, leaded to ectopic lipid accumulation in the liver and BAT, and decreased insulin signaling transduction in mice. Obesity or high-lipid loading decreased BAP31 expression, which prevented the adipogenesis process and lipid accumulation in WAT, worsening obesity-induced lipid dysfunction. BAP31-deficiency reduces lipolysis and increases LDs expansion in adipocytes, even worsens insulin signaling, forming a vicious circle in adipocytes. We currently reported the dual roles of BAP31 in lipid metabolism via modulating adipogenesis and lipolysis in adipocytes, pointing the importance of proper expression of BAP31 in maintaining lipid homeostasis in WAT.

LDs are phylogenetically conserved organelles, with a unique physical structure: consisting of a hydrophobic core of neutral lipids, and capsuling by a phospholipid monolayer that is decorated by a diverse proteome 41. Different LDs in a cell contain different protein composition 42, and have different rates of acquiring TAG. Change in lipid composition of LDs has been implicated in numerous physiological and pathophysiological functions, including cancer 43, obesity, fatty liver, and neurodegeneration disorders 44. Exposure of phosphatidylserine (PS) on the plasma membrane is widely observed during cellular apoptosis, contributes to the recognition and subsequent removal of apoptotic bodies by phagocytes, and providing a binding site for the annexin V for apoptotic cell detecting 45. BAP31-deficiency increased PS content, as well as PL, LPC, PCp, and PI content in WAT of BAP31-ASKO mice (Figure 3). The function of BAP31 on membrane PS composition and the related apoptosis induction should be warranted in future studies. BAP31-deficiency promoted long chain PUFA-enriched TAG content in WAT (Figure 3), and is consistent with the previous observations from obese murine model and human species 46. Also, BAP31-deficiency increased PC/PE ratio. Obesity is associated with increased *de novo* PC synthesis, promoted PC turnover, and induced pro-inflammatory activation of adipose tissue macrophages 47. We determined increased expression of macrophage marker of Mcp1 in BAP31-ASKO mice upon HFD-feeding, suggesting that the increased PC/PE ratio may be involved in the increased pro-inflammation in WAT. BAP31-deficiency promoted most PLs content in WAT of mice, which are mostly localized in cell membrane. The composition is highly associated with molecular transportation, apoptosis, and signaling transduction 48, suggesting that BAP31 may modulate PLs composition and subsequently change the related biological reactions.

Overall, we reported the dual role of BAP31 in regulating lipid metabolism. BAP31-deficiency inhibited lipid accumulation via suppressing adipogenesis in WAT; prevented Plin1 degradation and promoted LDs abnormal growth, which reduced the lipolysis process and leaded to adipocyte expansion in mice.

## Supplementary Material

Supplementary figures and tables.

## Figures and Tables

**Table 1:** Serum metabolites of WT and BAP31-ASKO mice with food deprivation for 24 hours (unit: mM).

	WT-Fed	BAP31-ASKO-Fed	WT-Fasted	BAP31-ASKO-Fasted
Glucose	7.36±0.28	8.69±0.65^*^	1.72±0.25^&^	2.38±0.18^#^
TAG	0.51±0.06	0.84±0.09^*^	0.79±0.07^&^	0.63±0.05^#^
FFA	0.76±0.05	0.60±0.03^*^	1.44±0.03^&^	1.27±0.06^#^
Cholesterol	5.15±0.26	4.72±0.13	5.94±0.34^&^	5.71±0.18
Glycerol	0.44±0.01	0.41±0.00^*^	0.47±0.00^&^	0.43±0.00^#^

WT and BAP31-ASKO mice were food deprived with free access to water for 24 hours, then were sacrificed under anesthesia. Blood was extracted and sera were purified. Serum metabolites were determined using the commercial kits. n=6-7. *p<0.05, compared to WT-Fed mice; *^&^p*<0.05, compared to WT-Fed mice. ^#^*p*<0.05, compared to BAP31-ASKO-Fed mice.

**Table 2:** Serum metabolites of mice treated with CL316,243 for 1 hour (unit: mM).

	WT-CL	BAP31-ASKO-CL
Glucose	4.95±0.36	5.96±0.39*
TAG	0.74±0.05	0.67±0.05
FFA	0.58±0.04	0.45±0.06*
Glycerol	0.47±0.01	0.44±0.01*

WT and BAP31-ASKO mice (12-week-old) were injected with CL316,243 (CL, 0.1 mg/kg BW) intraperitoneally. One hour later, mice were sacrificed under anesthesia. Blood was extracted and sera were purified. Serum metabolites were determined using the commercial kits. n=7. *p<0.05, compared to WT-CL mice.

## Abbreviations

BAP31: B-cell receptor-associated protein 31
BW: body weight
Cebpα: CCAAT enhancer-binding protein alpha
Chop: C/EBP homologous protein
CHX: cycloheximide
CQ: chloroquine
Co-IP: Co-immunoprecipitation
ER: endoplasmic reticulum
Fabp4: fatty acid binding protein 4
Fas: fatty acid synthase
FFA: free-fatty acids
Fsp27: fat-specific protein 27
Grp78: glucose-regulated protein 78
H/E: hematoxylin and eosin
Hsl: hormone-sensitive lipase
ISO: isoproterenol
LDs: lipid droplets
Lpl: lipoprotein lipase
MEFs: mouse embryonic fibroblasts
Mgl: monoglyceride lipase
OA: oleic acid
PC: phosphatidylcholines
PDI: protein disulfide isomerase
PE: phosphatidylethanolamine
PI: phosphatidylinositols
Plin1: perilipin1
Pparγ: peroxisome proliferator-activated receptor γ
PS: phosphatidylserine
TAG: triglycerides
WAT: white adipose tissue

## References

[1] Reitman ML (2021). How does obesity promote breast cancer tumor growth?. Cell Metab.

[2] Wang QA, Tao C, Gupta RK, Scherer PE (2013). Tracking adipogenesis during white adipose tissue development, expansion and regeneration. Nat Med.

[3] Olzmann JA, Carvalho P (2019). Dynamics and functions of lipid droplets. Nat Rev Mol Cell Biol.

[4] Sun Z, Gong J, Wu L, Li P (2013). Imaging lipid droplet fusion and growth. Methods Cell Biol.

[5] Miyoshi H, Souza SC, Zhang HH, Strissel KJ, Christoffolete MA, Kovsan J (2006). Perilipin promotes hormone-sensitive lipase-mediated adipocyte lipolysis via phosphorylation-dependent and -independent mechanisms. J Biol Chem.

[6] Tansey JT, Sztalryd C, Gruia-Gray J, Roush DL, Zee JV, Gavrilova O (2001). Perilipin ablation results in a lean mouse with aberrant adipocyte lipolysis, enhanced leptin production, and resistance to diet-induced obesity. Proc Natl Acad Sci U S A.

[7] Sun Z, Gong J, Wu H, Xu W, Wu L, Xu D (2013). Perilipin1 promotes unilocular lipid droplet formation through the activation of Fsp27 in adipocytes. Nat Commun.

[8] Brasaemle DL, Barber T, Kimmel AR, Londos C (1997). Post-translational regulation of perilipin expression. Stabilization by stored intracellular neutral lipids. J Biol Chem.

[9] Xu G, Sztalryd C, Londos C (2006). Degradation of perilipin is mediated through ubiquitination-proteasome pathway. Biochim Biophys Acta.

[10] Dalen KT, Schoonjans K, Ulven SM, Weedon-Fekjaer MS, Bentzen TG, Koutnikova H (2004). Adipose tissue expression of the lipid droplet-associating proteins S3-12 and perilipin is controlled by peroxisome proliferator-activated receptor-gamma. Diabetes.

[11] Kim KM, Adachi T, Nielsen PJ, Terashima M, Lamers MC, Kohler G (1994). Two new proteins preferentially associated with membrane immunoglobulin D. EMBO J.

[12] Chandra D, Choy G, Deng X, Bhatia B, Daniel P, Tang DG (2004). Association of active caspase 8 with the mitochondrial membrane during apoptosis: potential roles in cleaving BAP31 and caspase 3 and mediating mitochondrion-endoplasmic reticulum cross talk in etoposide-induced cell death. Mol Cell Biol.

[13] Dang E, Yang S, Song C, Jiang D, Li Z, Fan W (2018). BAP31, a newly defined cancer/testis antigen, regulates proliferation, migration, and invasion to promote cervical cancer progression. Cell Death Dis.

[14] Abe F, Van Prooyen N, Ladasky JJ, Edidin M (2009). Interaction of Bap31 and MHC class I molecules and their traffic out of the endoplasmic reticulum. J Immunol.

[15] Niu K, Xu J, Cao Y, Hou Y, Shan M, Wang Y (2017). BAP31 is involved in T cell activation through TCR signal pathways. Sci Rep.

[16] Wang B, Heath-Engel H, Zhang D, Nguyen N, Thomas DY, Hanrahan JW (2008). BAP31 interacts with Sec61 translocons and promotes retrotranslocation of CFTRDeltaF508 via the derlin-1 complex. Cell.

[17] Calhoun AR, Raymond GV (2014). Distal Xq28 microdeletions: clarification of the spectrum of contiguous gene deletions involving ABCD1, BCAP31, and SLC6A8 with a new case and review of the literature. Am J Med Genet A.

[18] Li G, Jiang X, Liang X, Hou Y, Zang J, Zhu B (2023). BAP31 regulates the expression of ICAM-1/VCAM-1 via MyD88/NF-kappaB pathway in acute lung injury mice model. Life Sci.

[19] Iwasa M, Yamagata T, Mizuguchi M, Itoh M, Matsumoto A, Hironaka M (2013). Contiguous ABCD1 DXS1357E deletion syndrome: report of an autopsy case. Neuropathology.

[20] Ernst WL, Shome K, Wu CC, Gong X, Frizzell RA, Aridor M (2016). VAMP-associated Proteins (VAP) as Receptors That Couple Cystic Fibrosis Transmembrane Conductance Regulator (CFTR) Proteostasis with Lipid Homeostasis. J Biol Chem.

[21] Xu JL, Li LY, Wang YQ, Li YQ, Shan M, Sun SZ (2018). Hepatocyte-specific deletion of BAP31 promotes SREBP1C activation, promotes hepatic lipid accumulation, and worsens IR in mice. J Lipid Res.

[22] Wu Z, Yang F, Jiang S, Sun X, Xu J (2018). Induction of Liver Steatosis in BAP31-Deficient Mice Burdened with Tunicamycin-Induced Endoplasmic Reticulum Stress. Int J Mol Sci.

[23] Xu J, Kulkarni SR, Donepudi AC, More VR, Slitt AL (2012). Enhanced Nrf2 activity worsens insulin resistance, impairs lipid accumulation in adipose tissue, and increases hepatic steatosis in leptin-deficient mice. Diabetes.

[24] Heckmann BL, Zhang X, Xie X, Saarinen A, Lu X, Yang X (2014). Defective adipose lipolysis and altered global energy metabolism in mice with adipose overexpression of the lipolytic inhibitor G0/G1 switch gene 2 (G0S2). J Biol Chem.

[25] Cybulski N, Polak P, Auwerx J, Ruegg MA, Hall MN (2009). mTOR complex 2 in adipose tissue negatively controls whole-body growth. Proc Natl Acad Sci U S A.

[26] Song JW, Lam SM, Fan X, Cao WJ, Wang SY, Tian H (2020). Omics-Driven Systems Interrogation of Metabolic Dysregulation in COVID-19 Pathogenesis. Cell Metab.

[27] Przygrodzka E, Hou X, Zhang P, Plewes MR, Franco R, Davis JS (2021). PKA and AMPK Signaling Pathways Differentially Regulate Luteal Steroidogenesis. Endocrinology.

[28] Namba T (2019). BAP31 regulates mitochondrial function via interaction with Tom40 within ER-mitochondria contact sites. Sci Adv.

[29] London E, Bloyd M, Stratakis CA (2020). PKA functions in metabolism and resistance to obesity: lessons from mouse and human studies. J Endocrinol.

[30] Wolins NE, Quaynor BK, Skinner JR, Schoenfish MJ, Tzekov A, Bickel PE (2005). S3-12, Adipophilin, and TIP47 package lipid in adipocytes. J Biol Chem.

[31] Spiliotis ET, Manley H, Osorio M, Zuniga MC, Edidin M (2000). Selective export of MHC class I molecules from the ER after their dissociation from TAP. Immunity.

[32] Annaert WG, Becker B, Kistner U, Reth M, Jahn R (1997). Export of cellubrevin from the endoplasmic reticulum is controlled by BAP31. J Cell Biol.

[33] Ducret A, Nguyen M, Breckenridge DG, Shore GC (2003). The resident endoplasmic reticulum protein, BAP31, associates with gamma-actin and myosin B heavy chain. Eur J Biochem.

[34] Thompson KE, Bashor CJ, Lim WA, Keating AE (2012). SYNZIP protein interaction toolbox: *in vitro* and *in vivo* specifications of heterospecific coiled-coil interaction domains. ACS Synth Biol.

[35] Zhao X, Gao M, He J, Zou L, Lyu Y, Zhang L (2015). Perilipin1 deficiency in whole body or bone marrow-derived cells attenuates lesions in atherosclerosis-prone mice. PLoS One.

[36] Tang QQ, Otto TC, Lane MD (2003). Mitotic clonal expansion: a synchronous process required for adipogenesis. Proc Natl Acad Sci U S A.

[37] Yuan Q, Zhao B, Cao YH, Yan JC, Sun LJ, Liu X (2022). BCR-Associated Protein 31 Regulates Macrophages Polarization and Wound Healing Function via Early Growth Response 2/C/EBPbeta and IL-4Ralpha/C/EBPbeta Pathways. J Immunol.

[38] Petersen RK, Madsen L, Pedersen LM, Hallenborg P, Hagland H, Viste K (2008). Cyclic AMP (cAMP)-mediated stimulation of adipocyte differentiation requires the synergistic action of Epac- and cAMP-dependent protein kinase-dependent processes. Mol Cell Biol.

[39] Marion-Letellier R, Savoye G, Ghosh S (2016). Fatty acids, eicosanoids and PPAR gamma. Eur J Pharmacol.

[40] Farese RV Jr, Walther TC (2016). Lipid droplets go nuclear. J Cell Biol.

[41] Wolins NE, Brasaemle DL, Bickel PE (2006). A proposed model of fat packaging by exchangeable lipid droplet proteins. FEBS Lett.

[42] Ducharme NA, Bickel PE (2008). Lipid droplets in lipogenesis and lipolysis. Endocrinology.

[43] Petan T (2020). Lipid Droplets in Cancer. Rev Physiol Biochem Pharmacol.

[44] Farmer BC, Walsh AE, Kluemper JC, Johnson LA (2020). Lipid Droplets in Neurodegenerative Disorders. Front Neurosci.

[45] Lee SH, Meng XW, Flatten KS, Loegering DA, Kaufmann SH (2013). Phosphatidylserine exposure during apoptosis reflects bidirectional trafficking between plasma membrane and cytoplasm. Cell Death Differ.

[46] Lange M, Angelidou G, Ni Z, Criscuolo A, Schiller J, Bluher M (2021). AdipoAtlas: A reference lipidome for human white adipose tissue. Cell Rep Med.

[47] Petkevicius K, Virtue S, Bidault G, Jenkins B, Cubuk C, Morgantini C (2019). Accelerated phosphatidylcholine turnover in macrophages promotes adipose tissue inflammation in obesity. Elife.

[48] Groves JT, Kuriyan J (2010). Molecular mechanisms in signal transduction at the membrane. Nat Struct Mol Biol.

