# Supplementary material for: BAP31 depletion inhibited adipogenesis, repressed lipolysis and promoted lipid droplets abnormal growth via attenuating Perilipin1 proteasomal degradation
Source: Int J Biol Sci. 2023 Mar 13;19(6):1713–30. doi: 10.7150/ijbs.82178 (PMC10092757; doi:10.7150/ijbs.82178)
Supplement: Supplementary file 1 — Supplementary figures and tables. [file ijbsv19p1713s1.pdf]

Supporting documents/data

Supplementary Figure. S1. BAP31-deficiency reduced the transcriptional levels of adipogenic markers of Cebpα, Cebpβ, Pparγ, Fabp4, Lpl, and Adiponection in mouse embryonic fibroblasts. MEFs from BAP31$^{flox/-}$ mice were infected with Ad-LacZ and Ad-Cre were induced to differentiation to adipocytes. Total RNA was extracted from differentiated MEFs 0, 1, 3, 5, and 7 days post differentiation. The transcriptional levels of Cebpα, Cebpβ, Pparγ, Fabp4, Lpl, and Adiponection were determined via quantitative real-time PCR. *p<0.05, compared to Ad-LacZ MEFs.

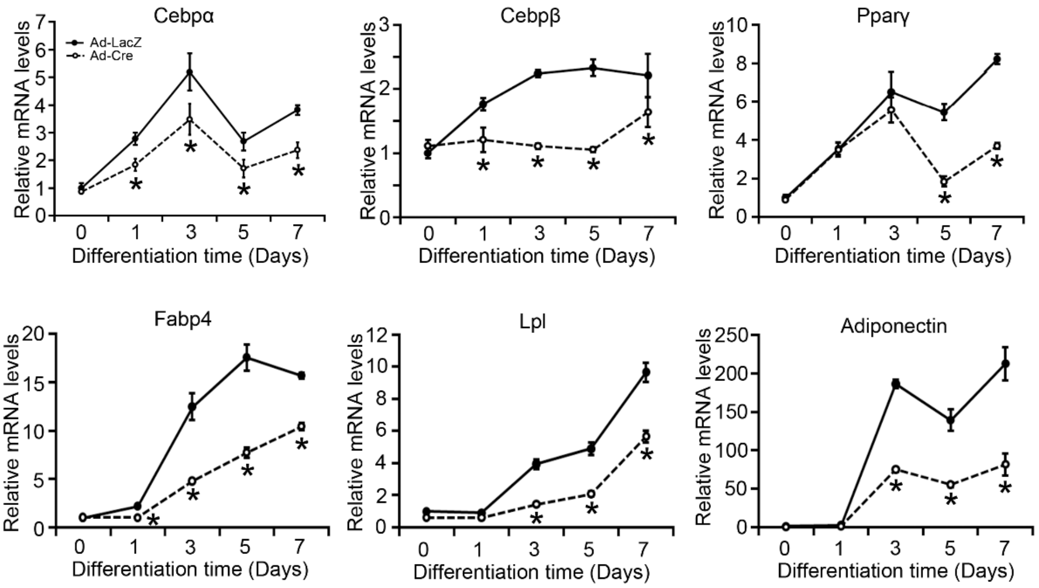

Supplementary Figure. S2. BAP31-deficiency in adipose tissue fail to reduced fasting-induced PKA activation and Atgl expression in liver tissues. WT and BAP31-ASKO mice were food deprived for 24 hours. Liver tissues were dissected and the protein levels of p-PKA, PKA, p-Hsl (563), Hsl, Atgl, Plin1, and BAP31 were determined. GAPDH was used as the loading control.

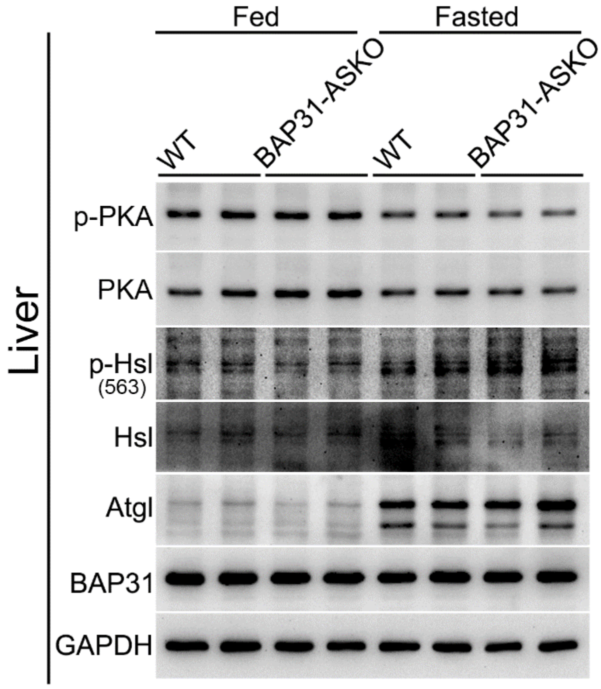

Supplementary Figure. S3. The total lipase activity in epididymal (Epi) WAT, quadriceps muscles, liver tissues and serum were determined. Lipase activity was detected using the commercial kits from Nanjing Jiancheng Biomedical Company (Nanjing, China). Tissues were homogenized with the lysis buffer which kits supplied. And then the activity of the lysates and serum were measured spectrophotometrically. n=6-7. *p<0.05, compared to WT controls.

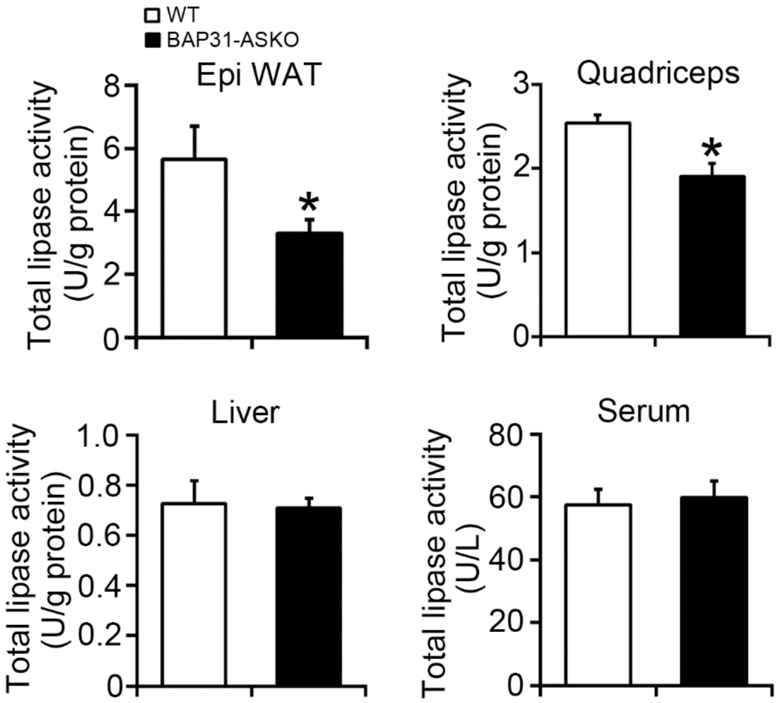

Supplementary Table S1. Primer sequences for quantitative real-time PCR.

Genes	Forward	Reverse
18S	AGTCCCTGCCCTTTGTACACA	CGATCCGAGGGCCTCACTA
Adiponectin	TGTTCCTCTTAATCCTGCCCA	CCAACCTGCACAAGTTCCCTT
Atgl	GGATGGCGGCATTTCAGACA	CAAAGGGTTGGGTTGGTTCAG
BAP31	GCCACCTTCCTCTACGCAG	TGCCATAGGTCACTACCAACTC
Cebpα	CAAGAACAGCAACGAGTACCG	GTCACTGGTCAACTCCAGCAC
Cebpβ	GGACTACGCAACACACGTGTAACT	ACAAAACCAAAAACATCAACAACC
Ccl3	TTCTCTGTACCATGACACTCTGC	CGTGGAATCTTCCGGCTGTAG
Fabp4	AAGGTGAAGAGCATCATAACCCT	TCACGCCTTTCATAACACATTCC
Hsl	GATGTCACAGTCAATGGAGACAC	GGTGAAACCCCTCAGGGAAAG
Lpl	GGGAGTTTGGCTCCAGAGTTT	TGTGTCTTCAGGGGTCCTTAG
Mcp1	TTAAAAACCTGGATCGGAACCAA	GCATTAGCTTCAGATTTACGGGT
Mgl	CAGAGAGGCCAACCTACTTTTC	ATGCGCCCCAAGGTCATATTT
Pparγ	CTCTGTTTTATGCTGTTATGGGTGA	GGTCAACAGGAGAATCTCCCAG
Si-BAP31	GGUUCUCAUCGUCAUCCUUTT	AAGGAUGACGAUGAGAACCTT

Supplemental Table S2: List of antibodies used in this study

Antibody	Isotype	Cat#	Source	Dilution
Adiponectin	Rabbit IgG	2789	CST	1:1000
Atgl	Rabbit IgG	2138	CST	1:1000
BAP31	Rabbit IgG	4043	CST	1:1000
β-Actin	Mouse IgG	60008-1-Ig	Proteintech	1:5000
Cebpα	Rabbit IgG	2295	CST	1:1000
CHOP	Rabbit IgG	2895	CST	1:1000
Fabp4	Rabbit IgG	2120	CST	1:1000
Fas	Rabbit IgG	3180	CST	1:2000
Flag	Rabbit IgG	20543-1-AP	Proteintech	1:2000
GAPDH	Rabbit IgG	2118	CST	1:5000
HA	Rabbit IgG	51064-2-AP	Proteintech	1:5000
HSL	Rabbit IgG	4107	CST	1:1000
MCP1	Mouse IgG	66272-1-Ig	Proteintech	1:1000
PDI	Rabbit IgG	3501	CST	1:1000
Perilipin 1	Rabbit IgG	9349	CST	1:1000
p-HSL (563)	Rabbit IgG	4139	CST	1:1000
p-HSL (565)	Rabbit IgG	4137	CST	1:1000
PKA	Rabbit IgG	4782	CST	1:1000
p-PKA	Rabbit IgG	9621	CST	1:1000
PPARγ	Rabbit IgG	2443	CST	1:2000
Ubiquitin	Mouse IgG	3936	CST	1:1000

CST: cell signaling technology. Proteintech: Proteintech Group, Inc.

Supplementary table S3: Organ indexes of WT and BAP31-ASKO mice at 20- and 50-week-old age.

	20-week-old		50-week-old	
	WT	BAP31-ASKO	WT	BAP31-ASKO
Liver	1.47±0.05	1.53±0.07	1.47±0.04	1.60±0.11*
Liver/BW (%)	5.23±0.08	5.58±0.21	4.87±0.11	5.24±0.11*
Sk muscles	0.32±0.01	0.3±0.01	0.33±0.01	0.33±0.01
Sk muscles/BW (%)	1.13±0.03	1.1±0.03	1.08±0.02	1.09±0.02
BAT	0.16±0.01	0.18±0.01*	0.22±0.01	0.20±0.01
BAT/BW (%)	0.58±0.03	0.67±0.03*	0.71±0.04	0.66±0.03

WT and BAP31-ASKO at 20- and 50-week-old age were anesthetized and the organs were dissected. Liver, skeletal muscles of gastrocnemius and quadriceps, and brown adipose tissue were weighted. n=8-10. *p<0.05, compared to WT mice.
